# A simple nonradioactive method for the determination of the binding affinities of antibodies induced by hapten bioconjugates for drugs of abuse

**DOI:** 10.1007/s00216-015-9223-z

**Published:** 2015-12-16

**Authors:** Oscar B. Torres, Joshua F. G. Antoline, Fuying Li, Rashmi Jalah, Arthur E. Jacobson, Kenner C. Rice, Carl R. Alving, Gary R. Matyas

**Affiliations:** Laboratory of Adjuvant and Antigen Research, US Military HIV Research Program, Walter Reed Army Institute of Research, 503 Robert Grant Avenue, Silver Spring, MD 20910 USA; U.S. Military HIV Research Program, Henry M. Jackson Foundation for the Advancement of Military Medicine, 6720A Rockledge Drive, Bethesda, MD 20817 USA; Drug Design and Synthesis Section, Molecular Targets and Medications Discovery Branch, National Institute on Drug Abuse, National Institutes of Health, Department of Health and Human Services, 9800 Medical Drive, Bethesda, MD 20892 USA; National Institute on Alcohol Abuse and Alcoholism, National Institutes of Health, 9800 Medical Drive, Bethesda, MD 20892 USA; WuXi AppTec (Shanghai) Co., Ltd, 288 FuTe Road, Waigaoqiao Free Trade Zone, Shanghai, 200131 China

**Keywords:** Heroin hapten, Competition ELISA, Equilibrium dialysis, UPLC/MS/MS, Apparent dissociation constant (*K*_d_), Antibody affinity

## Abstract

The accurate analytical measurement of binding affinities of polyclonal antibody in sera to heroin, 6-acetylmorphine (6-AM), and morphine has been a challenging task. A simple nonradioactive method that uses deuterium-labeled drug tracers and equilibrium dialysis (ED) combined with ultra performance liquid chromatography/tandem mass spectrometry (UPLC/MS/MS) to measure the apparent dissociation constant (*K*_d_) of antibodies to 6-AM and morphine is described. The method can readily detect antibodies with *K*_d_ in the low nanomolar range. Since heroin is rapidly degraded in sera, esterase inhibitors were included in the assay, greatly reducing heroin hydrolysis. MS/MS detection directly measured the heroin in the assay after overnight ED, thereby allowing the quantitation of % bound heroin in lieu of *K*_d_ as an alternative measurement to assess heroin binding to polyclonal antibody sera. This is the first report that utilizes a solution-based assay to quantify heroin-antibody binding without being confounded by the presence of 6-AM and morphine and to measure *K*_d_ of polyclonal antibody to 6-AM. Hapten surrogates 6-AcMorHap, 6-PrOxyHap, MorHap, DiAmHap, and DiPrOxyHap coupled to tetanus toxoid (TT) were used to generate high affinity antibodies to heroin, 6-AM, and morphine. In comparison to competition ED-UPLC/MS/MS which gave *K*_d_ values in the nanomolar range, the commonly used competition enzyme-linked immunosorbent assay (ELISA) measured the 50 % inhibition concentration (IC_50_) values in the micromolar range. Despite the differences in *K*_d_ and IC_50_ values, similar trends in affinities of hapten antibodies to heroin, 6-AM, and morphine were observed by both methods. Competition ED-UPLC/MS/MS revealed that among the five TT-hapten bioconjugates, TT-6-AcMorHap and TT-6-PrOxyHap induced antibodies that bound heroin, 6-AM, and morphine. In contrast, TT-MorHap induced antibodies that poorly bound heroin, while TT-DiAmHap and TT-DiPrOxyHap induced antibodies either did not bind or poorly bound to heroin, 6-AM, and morphine. This simple and nonradioactive method can be extended to other platforms, such as oxycodone, cocaine, nicotine, and methamphetamine for the selection of the lead hapten design during substance abuse vaccine development.

**Electronic supplementary material:**

The online version of this article (doi:10.1007/s00216-015-9223-z) contains supplementary material, which is available to authorized users.

**Graphical Abstract:**
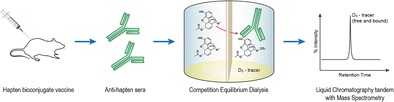
Determination of the antibody affinities using competition equilibrium dialysis (ED) combined with ultra performance liquid chromatography/tandem mass spectrometry (UPLC/MS/MS)

## Introduction

**Fig. 1:**
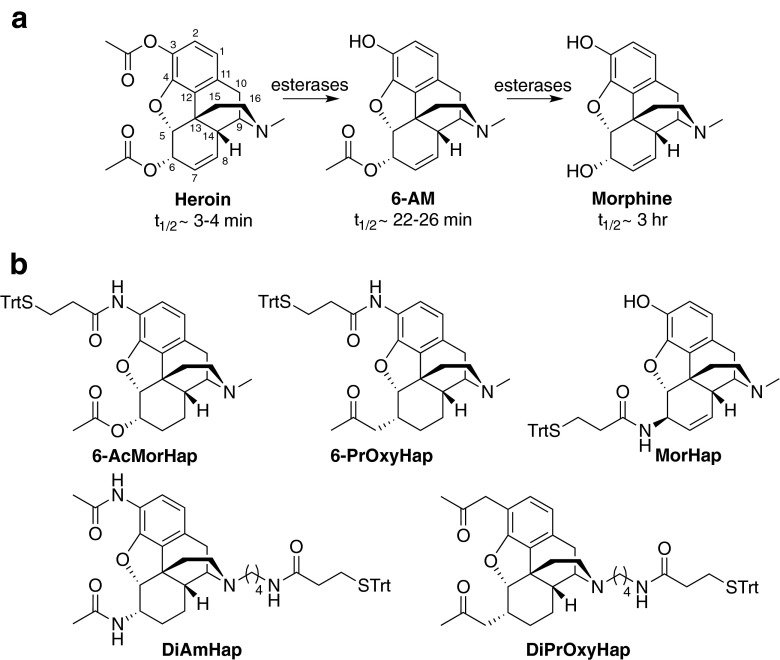
Heroin metabolites and heroin haptens. Degradation of heroin in humans (**a**). Heroin haptens with different linker attachment points (**b**). Haptens were coupled to tetanus toxoid (TT) to yield the TT-hapten bioconjugates

Therapeutic vaccines to drugs of abuse obstruct the psychoactive effects of the drugs by inducing antibodies that bind and prevent the drugs from traversing the blood-brain barrier [1, 2]. The development of a heroin vaccine presents a particular challenge because heroin rapidly metabolizes in serum to 6-acetylmorphine (6-AM) and morphine [3]. For effective sequestration of the drugs, hapten bioconjugate vaccines must induce both high titer and high affinity antibodies not only to heroin, but also to 6-AM and morphine [4, 5]. Vaccines to drugs of abuse have been increasingly explored as an alternative treatment for drug addiction. Vaccines have been efficacious in blunting the physiological effects of heroin and other abused drugs in animals. Several studies have demonstrated that the affinity and titer of the induced antibodies [1, 2] and hapten density [6, 7] of the hapten-protein conjugates are critical components of vaccine efficacy. There is a general agreement that enzyme-linked immunosorbent assay (ELISA) can readily and reliably quantify antibody titer [8–11]. In contrast, the measurement of hapten antibody affinity to heroin and its metabolites, as well as other substances of abuse, is generally estimated by competition ELISA [12, 13]. Concerns have been raised that competition ELISA does not accurately measure the antibody affinity [7, 14, 15]. Several of these concerns are as follows: (1) In serum, heroin is rapidly deacetylated to 6-AM and morphine by blood esterases (Fig. 1a) [16–20]. The enzymatic degradation of heroin during the assay changes the concentration of the drugs, and therefore, the calculated affinity constant may not be for heroin per se but for mixture of drugs. (2) The accuracy of ELISA is dependent on the hapten density of the coating agents [21]. (3) There is a localized high concentration of the hapten coated on the well, which allows for both arms of the immunoglobulin G (IgG) to bind to the well resulting in the requirement of high concentrations of competitive inhibitor to prevent antibody binding. (4) Similarly, the affinity of the antibody to the immunizing hapten is most likely higher than its affinity to the competitive inhibitor resulting in the further requirement of high concentrations of the competitive inhibitor. Based on these issues, a new methodology for the accurate measurement of the affinity of antibodies to drugs of abuse needs to be developed.

Equilibrium dialysis (ED) is the standard method for measuring the affinity of proteins to small molecules [22]. However, conventional ED between polyclonal sera and drugs cannot be employed to calculate the apparent dissociation constant (*K*_d_) because the antibody concentration is unknown. To circumvent this problem, a competition ED utilizing radioactive tracers as competitive inhibitors is often employed (Müller’s method) [23]. For the antibodies induced by heroin vaccines, the use of radioactive tracers to study heroin-antibody binding presents two main challenges. First, radioactive heroin and 6-AM are not commercially available. Second, the short half-life of heroin and its metabolites in sera is not compatible with the long equilibration time required for ED. We posit that (1) by using an alternative probe and detection system and (2) by identifying assay conditions that prevent the degradation of heroin and its metabolites during ED, binding affinities of heroin, 6-AM, and morphine to polyclonal antibody could be assessed.

**Fig. 2:**
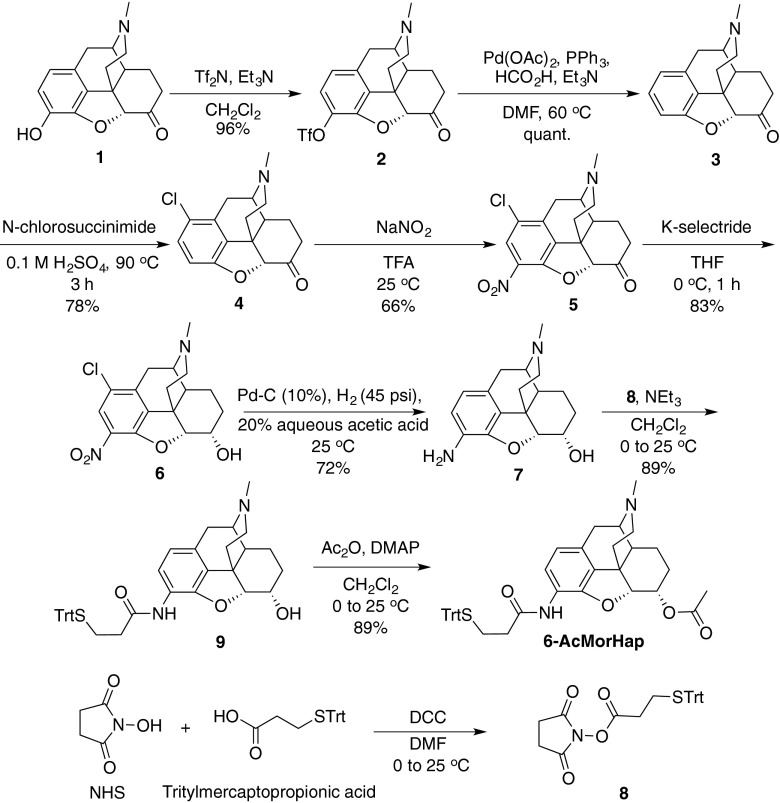
Synthesis of heroin hapten 6-AcMorHap

Liquid chromatography coupled with mass spectrometry is a valid approach for the detection of drug metabolites in biological fluids and could be used to quantify drugs that are bound to the antibody [24]. We rationalized that nonradioactive deuterium-labeled drug tracers (D_3_-tracers), such as D_3_-heroin, D_3_-6-AM, and D_3_-morphine, instead of radioactive analogs could be used as tracers in ED. In these D_3_-tracers, the methyl group of nitrogen is replaced by CD_3_ (see Electronic supplementary material (ESM) Fig. S1). Since the mass difference between the D_3_-tracer and corresponding unlabeled drug is significant (Δmass = 3.02 amu), mass spectrometry will be able to discriminate these structurally similar molecules. From an analytical standpoint, the obvious advantage of using D_3_-tracer/liquid chromatography/mass spectrometry as a probe/detection system over radioactive tracer/scintillation counter and unlabeled drug/enzymatic colorimetric method (ELISA) is that the former can evaluate the integrity of the probe during the assay condition and thereby an unambiguous affinity constant can be determined. We herein report a nonradioactive competition ED coupled with ultra performance liquid chromatography tandem mass spectrometry (UPLC/MS/MS) method that we used to assess the accurate binding affinities of heroin hapten antibodies to 6-AM and morphine. Using this method, (1) we demonstrate that different heroin hapten designs can induce antibodies of varying affinities (Fig. 1b), and (2) we substantiate that the concerns raised about competition ELISA are valid and that the simple methodology of competition ED-UPLC/MS/MS should be used to more accurately measure substance abuse vaccine-induced antibody affinity for the abused substance.

## Materials and methods

Tritylmercaptopropionic acid (≥98 %) was acquired from Chem-Impex International (Wood Dale, IL, USA). Chemical reagents and solvents were ACS reagent grade, obtained from Sigma-Aldrich Chem. Co., and were used without further purification. N-hydroxysuccinimide (NHS, 98 %), acetic anhydride (Ac_2_O, ≥99 %), 4-(dimethylamino)pyridine (DMAP, ≥99 %), formic acid for mass spectrometry, tetraisopropyl pyrophosphoramide (iso-OMPA), and bis(4-nitrophenyl) phosphate (BNPP, 99 %) were purchased from Sigma-Aldrich (Saint Louis, MO, USA). Calibrated solutions (1 mg/mL) of heroin.HCl.H_2_O, D_3_-heroin.HCl, 6-AM.HCl, D_3_-6-AM.HCl, morphine.H_2_O, and D_3_-morphine that were used for ED were purchased from Lipomed (Cambridge, MA, USA). Calibrated solutions used were Certified Reference Materials. Optima™ LC/MS grade ammonium formate (NH_4_HCOO), methanol (MeOH), acetonitrile (ACN), and water (H_2_O) were purchased from Fischer Scientific (Suwanee, GA, USA). Anti-morphine antibody (ab1060) was purchased from Abcam (Cambridge, MA, USA). Single-use equilibrium dialysis plate with 48 inserts was purchased from Thermo Fisher Scientific (Waltham, MA, USA). An insert (dialysis cassette) contains a sample and a buffer chamber separated by a membrane with 12 K molecular weight cutoff. Screw neck total recovery vial with polytetrafluoroethylene/silicone septa was purchased from Waters (Cambridge, MA, USA). Dulbecco’s phosphate buffered saline (DPBS, 10 mM Na_2_HPO_4_, 1.8 mM KH_2_PO_4_, 2.7 mM KCl, 137 mM NaCl, pH 7.4) that was used for equilibrium dialysis was purchased from Quality Biological Inc. (Gaithersburg, MD, USA).

### Synthesis of 6-AcMorHap

6-AcMorHap was synthesized in eight steps (Fig. 2). Triftylated hydromorphone (2), 3-desoxydihydromorphinone (3), 1-chloro-3-desoxydihydromorphinone (4), and 1-chloro-3-nitro-dihydromorphinone (5) were synthesized sequentially from hydromorphone (1) using the procedure described by Li et al. [25]. The detailed synthesis and analytical characterization of 1-chloro-3-nitro-dihydromorphine (6), 3-amino-dihydromorphine (7), 3-tritylthiopropanamide-dihydromorphine (9), and 6-AcMorHap were described in the ESM. The structure of 6-AcMorHap was confirmed by its ^1^H and ^13^C nuclear magnetic resonance (NMR) spectra (see ESM Figs. S2 and S3).

### Equilibration of D_3_-morphine and morphine

In a competition ED, the competitive inhibitor is added on one side of the cassette (buffer chamber) and the tracer to the other side (sample chamber). The two chambers are allowed to reach equilibrium prior to measurements. Equilibration time is the incubation period where ∼50 % of competitive inhibitor and tracer are located in both the buffer and sample chambers. Morphine (300 μL, 1333.3 nM in DPBS) and D_3_-morphine (100 μL, 5 nM in DPBS) were added into the buffer and sample chambers, respectively. Triplicates were allotted for each time interval: 3, 6, 12, and 24 h. The dialysis plate was covered with sealing tape and incubated at 4 °C/300 rpm on a ThermoMixer® C apparatus (Eppendorf AG, Hamburg, Germany). At a given time point, equal volumes (90 μL) were pipetted from both the sample and buffer chambers. The samples were subsequently placed in separate total recovery vials containing 1 μL of 10 % aqueous formic acid for UPLC/MS/MS analysis. The % drug in the sample and buffer chambers was calculated using the formula:

$$ \%{\mathrm{drug}}_{\mathrm{sample}\kern0.2em \mathrm{or}\kern0.2em \mathrm{b}\mathrm{u}\mathrm{f}\kern-0.1em \mathrm{f}\mathrm{e}\mathrm{r}\kern0.2em \mathrm{chamber}}=\frac{{\left[\mathrm{drug}\right]}_{\mathrm{sample}\kern0.2em \mathrm{or}\kern0.2em \mathrm{b}\mathrm{u}\mathrm{f}\kern-0.1em \mathrm{f}\mathrm{e}\mathrm{r}\kern0.2em \mathrm{chamber}}}{{\left[\mathrm{drug}\right]}_{\mathrm{sample}\kern0.2em \mathrm{chamber}}+{\left[\mathrm{drug}\right]}_{\mathrm{buf}\kern-0.1em \mathrm{f}\mathrm{e}\mathrm{r}\kern0.2em \mathrm{chamber}}}\times 100 $$

where [drug]_sample chamber_ and [drug]_buffer chamber_ are the concentrations of the drug in the sample and buffer chambers, respectively.

### Stability of heroin and its metabolites

For accurate measurement of *K*_d_, the drugs must remain stable during the equilibration time. The stability of heroin and its metabolites was assessed at different dilutions of the mouse sera. An aliquot of the drug (5 nM, 400 μL in DPBS) was treated with different dilutions (1:25, 1:50, 1:100, 1:200, and 1:400) of mouse sera. The reaction mixture was incubated at 4 °C and the amount of drug was monitored at a given time interval using UPLC/MS/MS. The % remaining drug was calculated using the formula:

$$ \%\ \mathrm{remaining}\ \mathrm{drug}=\frac{{\left[\mathrm{drug}\right]}_t}{{\left[\mathrm{drug}\right]}_{\mathrm{initial}}}\times 100 $$

where [drug]_*t*_ is the concentration of the drug at time *t* and [drug]_initial_ is the initial concentration of the drug.

### Competition equilibrium dialysis of monoclonal antibody

Anti-morphine antibody (ab1060) is a mouse monoclonal antibody, supplied as a protein G purified antibody (∼2 mg/mL). The approximate molecular weight of the IgG is 155 kDa. Based on this information, the antibody concentration of the stock solution is ∼12.9 μM. ab1060 has a reported *K*_d_ value of 2 nM, which was determined by proprietary ultraviolet-visible (UV-VIS) absorption spectroscopy method that correlated absorbance with the concentration of the ligand-antibody complex [26]. Samples for equilibrium dialysis were prepared by diluting the stock solution in DPBS to yield 2.4, 4.8, and 9.6 nM. Inhibition studies were conducted in a single-use equilibrium dialysis plate with inserts following manufacturer’s instruction. A 100-μL aliquot of ab1060 (2.4, 4.8, and 9.6 nM) with a fixed amount of D_3_-morphine (Table 1) was pipetted into the sample chamber, and a 300-μL aliquot of the morphine solution in dialysis buffer was added to the buffer chamber (1:400, vide infra). Morphine, which is the competitive inhibitor, was prepared at different concentrations. The total volume of the solution in the insert is 400 μL. The dialysis plate was covered with sealing tape and incubated at 4 °C/300 rpm on a ThermoMixer® C apparatus. After 24 h, equal volumes (90 μL) were pipetted from both the sample and buffer chambers and placed in separate total recovery vials containing 1 μL of 10 % aqueous formic acid for UPLC/MS/MS analysis.

**Table 1:** Affinity of monoclonal antibody ab1060 to morphine at different concentrations of the antibody and D_3_-morphine

Antibody, nM^a^	D_3_-morphine, nM^a^	*b* values^b^	Dissociation constant (*K*_d_, nM)^c^
9.6	5.0	0.94 ± 0.014	0.11 ± 0.01
4.8	5.0	0.78 ± 0.012	0.72 ± 0.05
2.4	5.0	0.41 ± 0.007	1.96 ± 0.17^d^
2.4	2.5	0.86 ± 0.060	0.10 ± 0.06
2.4	10	0.36 ± 0.001	0.81 ± 0.07

^a^Initial concentration of binding partners

^b^Fraction of D_3_-morphine bound to the monoclonal antibody in the absence of inhibitor

^c^All *K*_d_ values were calculated using Müller’s equation and are the mean of triplicate determinations ± standard deviation

^d^The affinity that is closest to the reported *K*_d_ value of 2 nM

### Competition equilibrium dialysis of anti-hapten sera

The preparation of anti-hapten sera and the strategy for conventional and competition ED were described in the ESM. Week 0 and week 9 sera were considered as negative and anti-hapten sera, respectively. Anti-hapten sera solutions were prepared by diluting the sera in DPBS (1:400, 1:800, or 1:600). Dialysis buffer was prepared by diluting the corresponding negative sera in DPBS (1:400, 1:800, or 1:600). Competitive inhibitors with different initial concentrations were prepared in the dialysis buffer. For the test samples, anti-hapten sera/D_3_-tracer was run against the negative sera/inhibitors at the same sera dilution. A 100-μL aliquot of anti-hapten sera containing 5 nM D_3_-tracer was pipetted into the sample chamber, and a 300-μL aliquot of the competitive inhibitor solution was added to the buffer chamber. For negative controls, negative sera/D_3_-tracer was run against the negative sera at the same sera dilution. A 100-μL aliquot of negative sera containing 5 nM D_3_-tracer in dialysis buffer was pipetted into the sample chamber, and a 300-μL aliquot of the dialysis buffer was added to the buffer chamber. Equilibrium dialysis and preparation of samples for UPLC/MS/MS was performed as described above. In the absence of hapten antibodies (i.e., negative sera), there is no selective partitioning of the D_3_-tracer in the sample chamber as all negative controls have 0 % bound D_3_-tracer. After equilibrium, the total concentration of the D_3_-tracer ([*T*_t_]) for both test samples and negative controls was ∼1.25 nM.

### Determination of *b* values for 6-AM and morphine

Parameter *b* is the fraction of bound D_3_-tracer in the absence of competitive inhibitors. Anti-hapten sera/D_3_-tracer was run against the negative sera at the same sera dilution. Briefly, a 100-μL aliquot of anti-hapten sera containing 5 nM D_3_-tracer was pipetted into the sample chamber and dialyzed against 300 μL of the appropriate dialysis buffer (1:25, 1:50, 1:100, 1:200, 1:400, 1:800, 1:1600). Equilibrium dialysis and preparation of samples for UPLC/MS/MS was performed as described above. The calculation of *b* values is discussed (vide infra) as part of the *K*_d_ calculation (see ESM Tables S1–S3).

### Determination of % bound heroin

Since the hydrolysis of heroin in diluted sera was extensive, the assay was performed in the presence of esterase inhibitors. All sera were diluted in DPBS (1:400) and pretreated with esterase inhibitors (iso-OMPA, 100 μM and BNPP, 100 μM) for 1 h at 4 °C. Briefly, a 100-μL aliquot of pretreated dilute sera with 5 nM heroin was pipetted into the sample chamber and dialyzed against 300 μL of the dialysis buffer (1:400). Equilibrium dialysis and preparation of samples for UPLC/MS/MS was performed as described above. The % bound heroin, which is the % heroin bound to the antibody in the presence of esterase inhibitors, was calculated using the equation:

$$ \%\ \mathrm{bound}\ \mathrm{heroin}=\frac{{\left[\mathrm{heroin}\right]}_{\mathrm{bound}}}{{\left[\mathrm{heroin}\right]}_{\mathrm{total}}}=\frac{{\left[\mathrm{heroin}\right]}_{\mathrm{sample}\ \mathrm{chamber}}-{\left[\mathrm{heroin}\right]}_{\mathrm{buffer}\ \mathrm{chamber}}}{{\left[\mathrm{heroin}\right]}_{\mathrm{sample}\ \mathrm{chamber}}}\times 100 $$

where [heroin]_sample chamber_ and [heroin]_buffer chamber_ are the concentrations of heroin in the sample and buffer chambers, respectively.

### UPLC/MS/MS

The UPLC conditions and mass spectrometry parameters were derived from Gottas et al. with some modifications [27]. The amount of drugs in the sample and buffer chambers was quantified using a Waters Acquity UPLC/TQD system equipped with an Acquity HSS T3 column (2.1 × 100 mm, 1.8 μm particle size) maintained at 65 °C. Samples were injected using full loop injection mode (10 μL) and ran against a 10-mM NH_4_HCOO pH 3.1/MeOH gradient (flow rate = 0.5 mL/min). The gradient profile is shown in ESM Table S4. The total cycle time was 8 min to ensure column equilibration prior to the next sample. In addition, a weak wash (600 μL, 10 % MeOH in H_2_O) and a strong wash (200 μL, 90 % ACN in H_2_O) were performed before each sample to prevent carryover.

Positive ionization was performed in multiple reaction monitoring (MRM) mode. The electrospray and source settings were as follows: 0.7 kV (capillary voltage), 120 °C (source temperature), 500 °C (desolvation temperature), 900 L/h (desolvation gas flow, N_2_), and 60 L/h (cone gas flow, N_2_). The collision gas (Ar) flow in the collision cell was maintained at 0.4 mL/min. The unlabeled drug and D_3_-tracer were differentiated based on their mass spectra (see ESM, Figs. S5–S10). Drugs were identified by comparing the retention times of the respective MRM transitions with the corresponding standards. Data were processed in the QuanLynx™ software and the drug concentrations in the sample and buffer chambers were quantified using peak area.

### Calculation of apparent dissociation constant (*K*_d_)

The *K*_d_ value can be calculated using Müller’s equation [23, 28]:

$$ {K}_{\mathrm{d}}=\left(\left[{I}_{50}\right]-\left[{T}_{\mathrm{t}}\right]\right)\left(1-1.5b+0.5{b}^2\right) $$

[*I*_50_], which is the molar concentration of the competitive inhibitor required for 50 % inhibition, was derived through interpolation on the % inhibition vs [*I*] curve. [*T*_t_] is the total molar concentration of the D_3_-tracer after equilibrium. The fraction of bound D_3_-tracer (*b*) in the absence of competitive inhibitor, *I*_0_, was calculated using the equation:

$$ b=\frac{{\left[{\mathrm{D}}_3\hbox{-} \mathrm{tracer}\right]}_{\mathrm{bound},\ {I}_0}}{{\left[{\mathrm{D}}_3\hbox{-} \mathrm{tracer}\right]}_{\mathrm{total},\ {I}_0}}=\frac{{\left[{\mathrm{D}}_3\hbox{-} \mathrm{tracer}\right]}_{\mathrm{sample}\ \mathrm{chamber}}-{\left[{\mathrm{D}}_3\hbox{-} \mathrm{tracer}\right]}_{\mathrm{buffer}\ \mathrm{chamber}}}{{\left[{\mathrm{D}}_3\hbox{-} \mathrm{tracer}\right]}_{\mathrm{sample}\ \mathrm{chamber}}} $$

where $\left[{\mathrm{D}}_3\hbox{-} \mathrm{tracer}\right]{}_{\mathrm{bound},\ {I}_0}$ is the concentration of the D_3_-tracer bound to the antibody in the absence of competitive inhibitor, $\left[{\mathrm{D}}_3\hbox{-} \mathrm{tracer}\right]{}_{\mathrm{total},\kern0.5em {I}_0}$ is the total (bound + free) concentration of the D_3_-tracer in the absence of competitive inhibitor, [D_3_-tracer]_sample chamber_ is the concentration of the D_3_-tracer in the sample chamber, and [D_3_-tracer]_buffer chamber_ is the concentration of the D_3_-tracer in the buffer chamber.

For tight-binding antibodies, the *b* values of the anti-hapten sera were from 0.4 to 0.7 even at high dilutions (1:400, 1:800, and 1:1600). No binding was assigned for anti-hapten sera with *b* values of ∼0 % at 1:25 dilution. The % inhibition at a given concentration of the competitive inhibitor, [*I*], was calculated using the equation:

$$ \begin{array}{c}\hfill \%\kern0.5em \mathrm{inhibition}=100\times \left(1-\frac{{\left[{\mathrm{D}}_3\hbox{-} \mathrm{tracer}\right]}_{\mathrm{bound},I}}{{\left[{\mathrm{D}}_3\hbox{-} \mathrm{tracer}\right]}_{\mathrm{bound},{I}_0}}\right)\hfill \\ {}\hfill {\left[{\mathrm{D}}_3\hbox{-} \mathrm{tracer}\right]}_{\mathrm{bound},I}={\left[{\mathrm{D}}_3\hbox{-} \mathrm{tracer}\right]}_{\mathrm{sample}\ \mathrm{chamber}}-{\left[{\mathrm{D}}_3\hbox{-} \mathrm{tracer}\right]}_{\mathrm{buffer}\ \mathrm{chamber}}\hfill \end{array} $$

where [D_3_-tracer]_bound, *I*_ is the concentration of the D_3_-tracer bound to the antibody in the presence of competitive inhibitor. The *K*_d_ was not determined at ≤1:100 dilution due to degradation of heroin, 6-AM, and morphine.

### ELISA and competition ELISA

The pooled mice sera were derived from previous animal studies [25, 29, 30]. ELISA was used to measure the serum antibody levels at week 9 against the heroin haptens. Competition ELISA was used to assess the cross-reactivity of the vaccine-induced anti-hapten sera to heroin and its major metabolites: 6-AM and morphine. ELISA and competition ELISA were performed as described previously [7]. The detailed procedures for ELISA and competition ELISA were provided in the ESM.

### Data analysis

Statistical analysis was performed using GraphPad Prism Version 6.0c. A *t* test (paired, two-tailed) was used to compare the concentration of the sample and buffer chambers. A one-way analysis of variance (ANOVA) with Tukey’s correction for multiple comparisons was used to compare the *K*_d_ values derived from different assay conditions and to compare the % intact heroin after 24 h from different assay conditions. A one-way ANOVA with anti-DiPrOxyHap as the control group and with Dunnett’s correction for multiple comparisons was used to compare the % bound heroin.

## Results

### Synthesis of heroin hapten 6-AcMorHap

6-AcMorHap was synthesized from hydromorphone 1 using a previously developed procedure for the introduction of the C-3 nitro group (Fig. 2) [25, 31]. Triflation of the C-3 phenol with *N*-phenyl-trifluoromethansulfonamide followed by palladium-mediated reductive cleavage afforded arene 3 in excellent yield. Direct C-3 nitration was stymied by undesired C-1 regioselectivity of the nitration. Instead, chlorination of the more reactive C-1 position with *N*-chlorosuccinimide allowed for selective C-3 nitration under mild conditions to give nitro compound 5 in moderate yield [32]. The C-6 ketone was reduced with K-selectride to give alcohol 6. Reduction of the nitro group was affected via palladium-catalyzed hydrogenation under acidic conditions, and it proceeded smoothly with concomitant cleavage of the C-1 chlorine to give amino alcohol 7. The aniline was selectively acylated with 2,5-dioxopyrrolidin-1-yl 3-(tritylthio)propanoate 8 to give amide 9 [33]. Finally, acylation of the hydroxyl moiety (Ac_2_O, DMAP) afforded heroin hapten 6-AcMorHap in 23 % over eight steps. The syntheses of 6-PrOxyHap, MorHap, DiAmHap, and DiPrOxyHap have been published [25, 30].

### Equilibration of D_3_-morphine and morphine

**Fig. 3:**
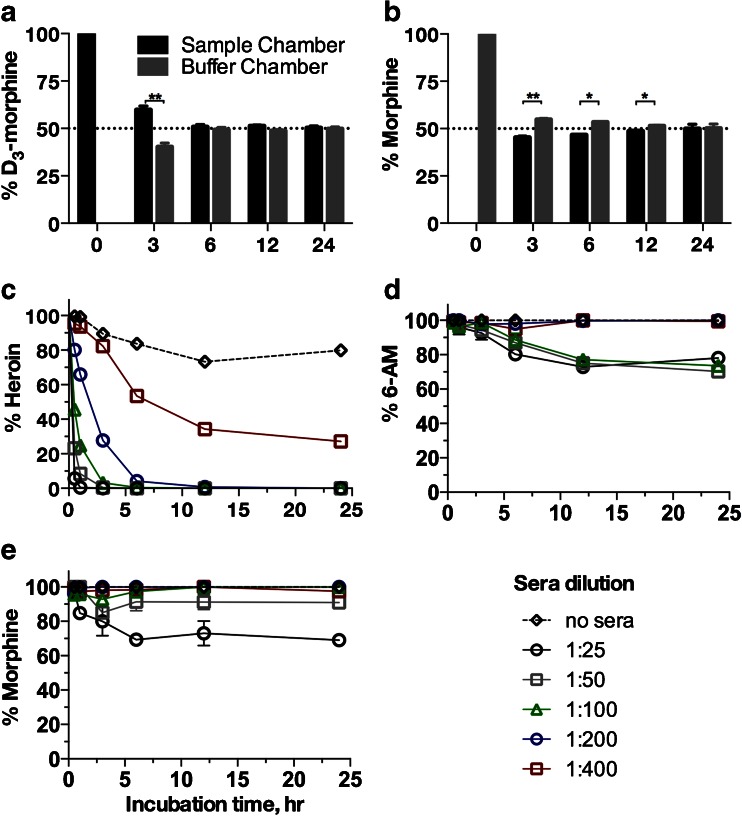
Equilibration of D_3_-morphine and morphine in dialysis cassettes (**a**, **b**). D_3_-morphine was added to one side (sample chamber) of the dialysis cassette and morphine to the other side (buffer chamber). D_3_-morphine and morphine concentrations reached equilibrium after 6 and 24 h, respectively. Drug concentrations between the two chambers were compared using paired *t* test (**p < 0.01, *p < 0.05). Stability of heroin, 6-AM, and morphine in the absence of sera or in the presence of sera at different sera dilutions in DPBS, pH 7.4 at 4 °C (**c**–**e**). Samples were incubated for the time indicated. No degradation of 6-AM and morphine was observed in the absence of sera. Partial heroin hydrolysis (∼20 %) was observed even in the absence of sera (also shown in Fig. 4a). Opiates were quantified by UPLC/MS/MS. Values are the mean of triplicate determinations ± standard deviation

The dialysis cassette consists of two chambers separated by a semipermeable membrane. The D_3_-tracer was added to the sample chamber while the competitive inhibitor was added to the buffer chamber. D_3_-morphine (5 nM, sample chamber) and morphine (1333.3 nM, buffer chamber) were allowed to diffuse in the semipermeable membrane (Fig. 3a, b). At 3 h, 59.68 ± 2.00 and 40.32 ± 2.00 % of D_3_-morphine partitioned into the sample and buffer chambers, respectively (Fig. 3a), and this difference between the % D_3_-morphine in the two chambers was significant (**p < 0.01, *t* test). There was no significant difference in D_3_-morphine concentrations between the sample and buffer chambers at 6, 12, and 24 h (*p* = not significant, *t* test). Also at 3 h, 45.32 ± 0.70 and 54.66 ± 0.70 % of morphine partitioned into the sample and buffer chambers, respectively (Fig. 3b, **p < 0.01, *t* test). The amount of morphine that partitioned into the sample chamber increased over time thereby decreasing the difference between the two chambers: 46.68 ± 0.17 % (6 h, *p < 0.05), 48.59 ± 0.39 % (12 h, *p < 0.05), and 49.97 ± 2.3 % (24 h, no significant difference compared to the buffer chamber). Based on these data, 24 h was selected as the standard time of incubation.

### Stability of the drugs in dilute sera

The degradation of heroin, 6-AM, and morphine was examined at different dilutions of the normal mouse sera for 24 h (Fig. 3c–e). There was an extensive hydrolysis of heroin at all dilutions (Fig. 3c). Thus, the calculation of antibody affinity against heroin was not performed. Hydrolysis of 6-AM was not observed at ≥1:200 dilutions (Fig. 3d). Degradation of morphine was not observed at ≥1:100 dilutions (Fig. 3e). Based on stability data and *b* values (vide infra), the calculation of antibody affinity against 6-AM and morphine was performed at ≥1:400 dilution.

### Apparent dissociation constant of a monoclonal antibody standard

The *K*_d_ of ab1060 at different concentrations of the antibody and the D_3_-morphine was determined (Table 1). Additionally, parameter *b*, defined as the fraction of the D_3_-tracer bound to the antibody in the absence of competitive inhibitor, was also considered for the assessment of *K*_d_ values. The *K*_d_ (1.96 ± 0.17 nM) derived from the assay condition with *b* = 0.41 was consistent with the reported *K*_d_ (2 nM) [34]. Although ED and UPLC/MS/MS have been individually considered as standard methods, this result confirms that the combination of competition ED-UPLC/MS/MS can be accurately used for drug-antibody binding quantification. A dramatic reduction in *K*_d_ was calculated when *b* values were outside of this range (Table 1). The results were consistent with the studies of Müller that the performance of competition ED must be done at assay conditions with *b* values of 0.4–0.7 [23].

### Opiate affinities of vaccine-induced antibodies by competition ED

For accurate determination of antibody affinity to 6-AM and morphine, the *K*_d_ was derived from different dilutions of pooled sera, which were obtained by immunization of the indicated hapten conjugated to TT and mixed with liposomal monophosphoryl lipid A as adjuvant (Table 2 and ESM Fig. S4) [25, 29, 30, 35]. In general, there was no statistical difference in *K*_d_ values when the assay conditions had a *b* value between 0.4 and 0.7 (ESM Tables S1 and S2). Antibodies to hapten conjugates 6-AcMorHap, 6-PrOxyHap, and MorHap had high affinities (*K*_d_ < 5 nM) to 6-AM and morphine (Table 2). Anti-6-AcMorHap had significantly higher affinities for both 6-AM and morphine relative to anti-6-PrOxyHap (*p* < 0.05, one-way ANOVA). Although anti-6-PrOxyHap and anti-MorHap had similar affinities to morphine, the affinity of anti-6-PrOxyHap to 6-AM was significantly higher than anti-MorHap (*p* < 0.01, one-way ANOVA). Both anti-DiAmHap and anti-DiPrOxyHap had low affinity for 6-AM and no affinity to morphine. In competition ED, low affinity and no binding were both determined in 1:25 sera dilution also to ensure that the lack of drug-antibody binding was not due to low concentration of the antibody, but due to the affinity of antibody for the drugs. Low affinity is defined as when >5 % of the D_3_-tracer was bound to the anti-hapten sera at a 1:25 sera dilution but was <5 % at 1:400. No binding is defined as when <5 % of the D_3_-tracer was bound to the anti-hapten sera at 1:25 sera dilution. Antibodies to DiAmHap and DiPrOxyHap bound 61.5 ± 2.3 and 9.1 ± 1.9 % D_3_-6-AM, respectively, at 1:25 sera dilution. However, the *K*_d_ cannot be accurately calculated because 6-AM was relatively unstable at 1:25 sera dilution (Fig. 3d). Both anti-DiAmHap and anti-DiPrOxyHap had 0 % binding to D_3_-morphine at all dilutions (ESM Tables S2 and S3). At 1:400 sera dilution, preimmune sera (week 0) do not bind D_3_-6-AM and D_3_-morphine, confirming that plasma proteins were not binding the opiates used in this study.

**Table 2:** *K*_d_ of pooled sera from mice immunized with different TT-hapten conjugates

Anti-hapten sera	Dissociation constant (*K*_d_, nM)^a^	
	6-AM	Morphine
6-AcMorHap	0.563 ± 0.05	0.555 ± 0.08
6-PrOxyHap	1.20 ± 0.19	1.40 ± 0.16
MorHap	2.94 ± 0.34	1.88 ± 0.46
DiAmHap	Low affinity^b^	No binding^c^
DiPrOxyHap	Low affinity^b^	No binding^c^

^a^All *K*_d_ values were calculated from the data shown in ESM Fig. S4 using Müller’s equation and are the mean of triplicate determinations ± standard deviation (see ESM, Tables S1 and S2). The affinities of anti-6-AcMorHap, anti-6-PrOxyHap, and anti-MorHap for 6-AM are different (multiple comparisons using one-way ANOVA). The affinities of anti-6-PrOxyHap and anti-MorHap for morphine are similar and are different to anti-AcMorHap (multiple comparisons using one-way ANOVA)

^b^>5 % of the D_3_-tracer was bound to the anti-hapten at 1:25 sera dilution but was <5 % at 1:400 dilution (see ESM, Table S1 and S3)

^c^<5 % of the D_3_-tracer was bound to the anti-hapten at 1:25 sera dilution (see ESM, Tables S2 and S3)

### Binding of heroin to vaccine-induced antibodies

**Fig. 4:**
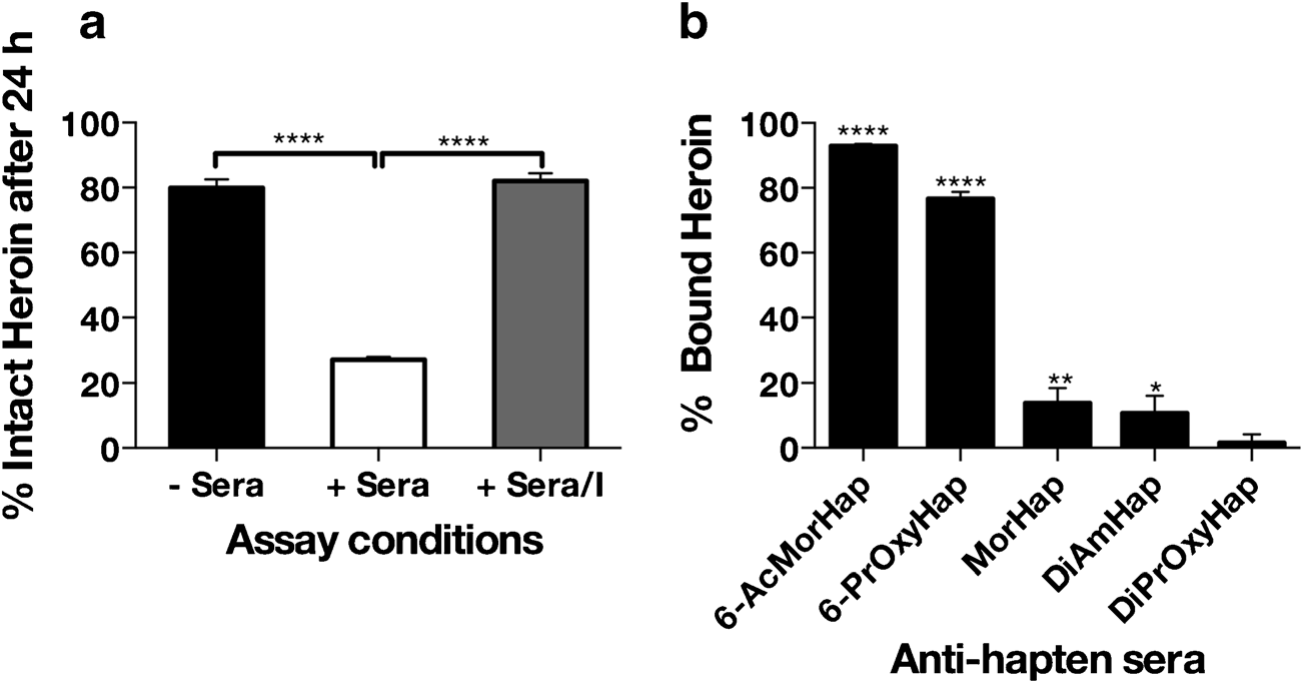
Assessment of the anti-hapten binding to heroin. Percent of intact heroin after 24 h in buffer with no sera (− Sera, DPBS), with sera (+ Sera, 1:400 sera dilution in DPBS), and with sera and inhibitors (+ Sera/*I*, 1:400 sera dilution in DPBS containing the esterase inhibitors iso-OMPA/BNPP) (**a**). Percent heroin in − Sera and + Sera/*I* was similar and different from + Sera (****p < 0.0001, multiple comparisons using one-way ANOVA). Percent of heroin that was bound to anti-hapten sera in the presence of iso-OMPA/BNPP (**b**). Percent bound heroin was different from anti-DiPrOxyHap sera (****p < 0.0001, **p < 0.01, *p < 0.05, multiple comparisons using one-way ANOVA). Opiates were quantified by UPLC-MS/MS. Sera from week 9 (3 weeks after two immunizations) from five mice per group were pooled and used in the assays. Values are the mean of triplicate determinations ± standard deviation

The *K*_d_ of polyclonal antibodies to heroin cannot be measured by competition ED-UPLC/MS/MS utilizing D_3_-heroin because of the extensive degradation of heroin in dilute sera. By extrapolation of the heroin stability data during ED with 1:400 sera dilution (Figs. 3c and 4a), heroin, D_3_-heroin, 6-AM, and D_3_-6-AM would be present at equilibrium with 6-AM and D_3_-6-AM as predominant chemical species. Although *K*_d_ cannot be accurately calculated, the assessment of heroin binding to polyclonal antibody is feasible if sufficient concentration of heroin is present in the assay. After 24 h, 79.9 ± 2.71 % heroin remained intact in DPBS (Fig. 4a). This observation was consistent with a previous report on the spontaneous hydrolysis of heroin at physiological pH [36]. This indicates that assay conditions must be identified such that the degradation of heroin in given serum dilution is comparable to spontaneous hydrolysis of heroin in buffer. The effect of inhibitors (*I*) on the stability of heroin was determined by measuring % intact heroin after 24 h in DPBS (− Sera), in dilute sera (+ Sera), and in dilute sera in the presence of OMPA/BNPP (+ Sera/*I*). The inhibitors did not suppress the spontaneous hydrolysis of heroin in buffer (*p* = not significant, one-way ANOVA) but did inhibit the extensive degradation of heroin in sera (****p < 0.0001, one-way ANOVA). The binding of heroin to anti-hapten sera was measured in the presence of inhibitors because the inhibitors blocked the serum esterase-mediated heroin degradation allowing for appreciable heroin concentrations that can be measured under these assay conditions. ED performed in the presence of these inhibitors showed that anti-6-AcMorHap, anti-6-PrOxyHap, anti-MorHap, and anti-DiAmHap bound significantly higher amount of heroin with 92.89 ± 0.72 % (****p < 0.0001), 76.66 ± 2.17 % (****p < 0.0001), 13.82 ± 4.66 % (**p < 0.01), and 10.75 ± 5.34 (*p < 0.05, one-way ANOVA), respectively, relative to anti-DiPrOxyHap (1.54 ± 2.66 %) (Fig. 4b, multiple comparisons using one-way ANOVA).

### Opiate affinities of vaccine-induced antibodies by competition ELISA

The most commonly used method for measuring the affinity of anti-hapten sera to small molecules has been competition ELISA because of its facile nature. In competitive ELISA, binding affinity is reported in terms of 50 % inhibition concentration (IC_50_). Analysis of the anti-hapten sera by competition ELISA indicated that anti-6-AcMorHap and anti-6-PrOxyHap had the highest relative affinities (IC_50_ < 10 μM) to heroin, 6-AM, and morphine (Table 3 and ESM Fig. S11). Anti-MorHap showed more modest affinity (IC_50_ ∼ 21 μM) to 6-AM, higher affinity for morphine (9.2 μM), and no affinity (IC_50_ > 10^3^ μM) to heroin. Anti-DiAmHap had no affinity for heroin and its metabolites. Anti-DiPrOxyHap displayed modest affinity to heroin, lower affinity to 6-AM (IC_50_ ∼ 145 μM), and no affinity to morphine. Comparison of these affinities with those obtained by competition ED-UPLC/MS/MS indicated that the affinity (IC_50_) derived from competition ELISA underestimated the actual *K*_d_ value by a factor of 10^3^–10^4^. Competition ELISA did not determine if the antibodies bind the drug of interest or if the degradation product was responsible for the binding (i.e., heroin degrading to 6-AM).

**Table 3:** Antibody titer and IC_50_ calculated using competition ELISA of pooled sera from mice immunized with different TT-hapten conjugates

Anti-hapten sera	Endpoint antibody titer^a^	Inhibition concentration (IC_50_, μM)^b^		
		Heroin	6-AM	Morphine
6-AcMorHap	819,200	0.5	1.1	0.1
6-PrOxyHap	409,600	2.2	7.5	1.6
MorHap	1,638,400	>1000	21.41	9.182
DiAmHap	204,800	>1000	>1000	>1000
DiPrOxyHap	102,400	16.8	144.9	>1000

^a^Binding ELISA was done against BSA-hapten coating antigen

^b^All IC_50_ values were calculated from competition ELISA data shown in ESM Fig. S11 using nonlinear regression log [inhibitor] vs. normalized response model

## Discussion

Drugs of abuse are too small to elicit antibody production and, therefore, require conjugation of structural analogs (haptens) to immunogenic proteins [37, 38]. Hapten bioconjugate vaccines are frequently used to generate functional antibodies against small molecules. The chemical structure of the hapten generally dictates the cross-reactivity of the anti-hapten sera, and thus, the hapten surrogate must be rationally designed [30, 39, 40]. To be efficacious, the hapten antibodies must cross-react with high affinity to the target drug(s).

**Fig. 5:**
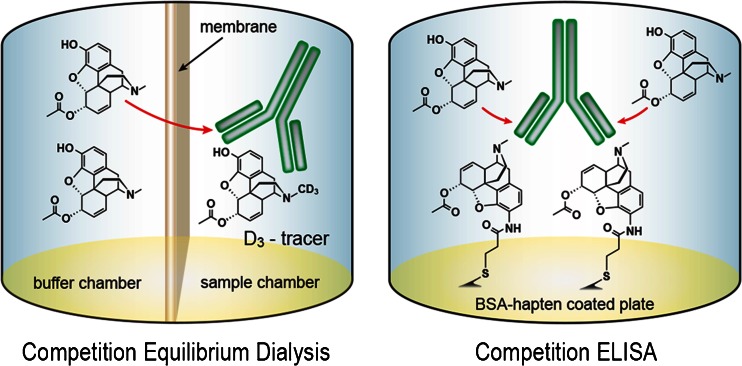
Comparison of competition ED and competition ELISA. In this example, the affinity of the anti-6-AcMorHap to 6-AM is measured. For competition ED, 6-AM is allowed to equilibrate with D_3_-6-AM/anti-6-AcMorHap. At equilibrium, the amount of drugs in both chambers is quantified by UPLC/MS/MS (*left panel*). For competition ELISA, the ELISA plate wells are precoated with bovine serum albumin (BSA)-6-AcMorHap and subsequently incubated with anti-6-AcMorHap. 6-AM is allowed to disrupt the BSA-6-AcMorHap/anti-6-AcMorHap interactions (*right panel*). The synthesis of BSA-6-AcMorHap was described in the ESM and Fig. S12

For heroin vaccines, the hapten design must mimic the key epitopes of heroin and its metabolites [4]. Currently, there has been no established rule that predicts whether a given hapten design would induce anti-hapten sera that target multiple chemical structures. To better understand the relationship between structure and cross-reactivity, the binding affinity of the antibodies induced by immunization with heroin haptens 6-AcMorHap, 6-PrOxyHap, MorHap, DiAmHap, and DiPrOxyHap conjugated to TT (Fig. 2) was assessed using competition ED-UPLC/MS/MS and competition ELISA (Fig. 5). We hypothesized that these hapten designs will induce antibodies with different cross-reactivities that will guide the future optimization of hapten structure. In terms of linker point attachment, the hapten design can be assigned into three groups: C3-linked haptens (6-AcMorHap and 6-PrOxyHap), C6-linked hapten (MorHap), and N-linked haptens (DiAmHap and DiPrOxyHap). The importance of linker point attachment was previously discussed by Matyas’ concept on facial recognition [30]. The conjugation of the haptenic surrogates to TT confines their freedom of motion, and thus, the hapten’s molecular structure bisects into two immunologically defined “faces.” The “front face” is defined as the key epitopes exposed to the immune system, while the “back face” is the sterically blocked moieties that cannot induce antibodies. Using the concept of facial recognition, 6-AcMorHap is particularly interesting because it preserves the C6-ester group that is the common moiety for both heroin and 6-AM [30]. Animals immunized with C3-linked hapten conjugates induced antibodies that bind morphine [41, 42]. In contrast to 6-AcMorHap, the haptens that were used by Spector and Koida contain a C6-alcohol group. We hypothesized that 6-AcMorHap has a “front face” that mimics heroin, 6-AM, and morphine, and thus, TT-6-AcMorHap bioconjugates will induce antibodies that cross-react with these three drugs (Fig. 1a, b) [30]. We also hypothesized that modification of an acetyl group to a propan-2-one group at the C6 position (i.e., 6-PrOxyHap) will reduce the binding affinity, but will not change the pattern of cross-reactivity because haptens 6-AcMorHap and 6-PrOxyHap have similar overall molecular shapes. Previously, we were not able to accurately measure the affinity of the anti-hapten sera due to the inherent limitations of competition ELISA [30]. Now that we can more accurately measure the affinity, we are able to demonstrate that our hypothesis of facial recognition has greater relevance [30].

In this study, competition ED utilizing D_3_-tracers has been developed to address the inherent constraints of competition ELISA. The main challenge in ED is to maintain the stability of the heroin and its metabolites during the assay. To address this problem, the equilibration time and the degradation of the drugs in dilute sera were investigated. Equilibration between the competitive inhibitor and the D_3_-tracer were attained at 24 h (Fig. 3a, b). However, this long equilibration time is not compatible with the short half-life of heroin and its metabolites in “neat” sera. We believed that by diluting the mouse sera, the concentration of the enzymes involved in heroin metabolism would be reduced. Consequently, the degradation of heroin and its metabolites would be suppressed. The degradation of the drugs in different dilutions of the sera was monitored for 24 h (Fig. 3c–e). At all dilutions, extensive degradation of heroin was observed. This suggests that the activity of the esterases, which are responsible for C3-ester group cleavage, cannot be suppressed by simple dilution. After 24 h, degradation of 6-AM and morphine was observed at lower dilutions but not at higher dilutions of sera. This indicates that the activity of the enzymes responsible for C6-ester group hydrolysis is concentration dependent. In general, morphine metabolism occurs in the liver. However, extrahepatic metabolism of morphine has been also reported [43]. The degradation profiles of the drugs seem to reach a plateau after 12 h, which might be due to loss of enzymatic activity. Collectively, ≥1:400 sera dilutions can be used to measure antibody affinities to 6-AM and morphine but not to heroin.

Another challenge that must be addressed in ED is the potential impact of plasma protein binding of the drugs. Recently, Zhang et al. reported the plasma protein binding of 222 drugs, of which 50 % showed 90–100 % binding [44]. In “neat” plasma/sera, heroin and morphine have ∼35 % plasma binding [45]. Presumably, 6-AM will also have ∼35 % plasma binding due to structural similarity to both drugs. If the binding of the D_3_-tracers to plasma protein is significant, the distinction of bonafide antibody-drug interactions from nonspecific protein binding becomes challenging. However, we believed that the effect of plasma binding could be reduced by sera dilution. At ≥1:400 dilution of the week 0 sera (i.e., preimmunized sera), D_3_-6-AM and D_3_-morphine showed ∼0 % binding to the plasma proteins. The binding of heroin to plasma proteins cannot be accurately assessed due to esterase-mediated degradation of heroin in dilute sera. However, extrapolation of the % bound heroin for anti-DiPrOxyHap sera conveys that the binding of heroin to plasma proteins is negligible in 1:400 sera dilution (Fig. 4b). Collectively, these data suggest that competition ED-UPLC/MS/MS can be used to measure antibody affinities to 6-AM and morphine in the dilute sera. For other drugs, where plasma protein binding is still significant even in dilute sera, the *K*_d_ values would difficult to calculate.

We tested the applicability of the competition ED-UPLC/MS/MS method for evaluating small drug-antibody interactions by measuring the dissociation constant of commercially available morphine monoclonal antibody ab1060. According to Müller, competition equilibrium dialysis should be carried out under the following conditions: (1) the *b* value of the antibody/tracer system should be ∼0.4–0.7, and (2) the specific radioactivity of the tracer should be optimal to prevent large deviations in the antibody affinity [23]. The effects of *b* values and D_3_-morphine concentrations on *K*_d_ were investigated using different assay conditions. Our results were consistent with the findings of Müller. A *K*_d_ of 1.96 nM, which is in agreement with the reported value of 2 nM [34], is obtained for ab1060 when *b* is 0.41 (Table 1 and ESM Fig. S4). At 2.4 nM ab1060/2.5 nM D_3_-morphine, a large deviation in *K*_d_ was observed. This most likely was because *b* was >0.7 and 2.5 nM D_3_-morphine was not an optimal tracer concentration. Considering the accuracy of affinity is dependent on both *b* values and specific concentration of the tracer, we utilized the *b* limit range of 0.4–0.7 and 5 nM D_3_-tracer for determining the *K*_d_ values of the anti-hapten sera.

Since antibody concentration is not required for the calculation of affinity, the competition ED-UPLC MS/MS could be extended to polyclonal sera [23]. As the polyclonal sera have high antibody titer (Table 3), we hypothesized that the concentration of hapten antibodies in dilute sera is still amenable for binding studies. The *b* values of anti-hapten sera were determined at both low and high sera dilutions (ESM Tables S1–S3). Anti-6-AcMorHap, anti-6-PrOxyHap, and anti-MorHap had *b* values of 0.4–0.7 at the range of 1:400–1:1600 dilutions. This suggests that *K*_d_ for 6-AM and morphine can be only calculated within a specific dilution range. At low sera dilution (≤1:100), 6-AM and morphine were prone to degradation, while at high sera dilution (≥1:1600, *b* < 0.4), the concentration of antibody was insufficient for reliable quantification. Anti-6-AcMorHap, anti-6-PrOxyHap, and anti-MorHap bound 6-AM and morphine with *K*_d_ values of <5 nM (Table 2 and ESM Fig. S4). Both anti-DiAmHap and anti-DiPrOxyHap have low affinity for 6-AM and no affinity for morphine. In contrast to the affinity values derived from ab1060, the *K*_d_ values of anti-hapten sera were not prone to large deviations when *b* ≥ 0.7. Based on the results, “pure” monoclonal antibody was more sensitive to variation in *K*_d_, when the *b* values were outside of the limit range. As the study of Müller was performed using anti-hapten polyclonal sera, the *b* limits of 0.4–0.7 might have limitations when applied to monoclonal antibodies. It is also possible that the optimal range of *b* values and D_3_-tracer concentrations vary from a given specific monoclonal antibody/drug system to another, and therefore, these values have to be determined empirically. In the case of the polyclonal anti-hapten sera that were used in this study, the *b* limit range of 0.4–0.7 was congruent with the findings of Müller, and the 5-nM D_3_-tracer concentration was optimal enough to prevent large deviation in *K*_d_ values.

To suppress the hydrolysis of heroin at 1:400 sera dilution, the specific esterase inhibitors iso-OMPA and BNPP were added to the reaction mixture [46–48]. The addition of iso-OMPA and BNPP increased the amount of intact heroin to ≥80 %, which was comparable to hydrolysis of heroin in DPBS in the absence of sera (Fig. 4a). This suggests that although *K*_d_ could not be calculated, the amount of % bound heroin to the antibody in lieu of *K*_d_ could be used to assess binding. Anti-6-AcMorHap and anti-6-PrOxyHap had 92.89 ± 0.72 and 76.66 ± 2.17 % of the heroin bound, respectively (Fig. 4b), suggesting that hapten designs 6-AcMorHap and 6-PrOxyHap can mimic crucial epitopes of heroin.

While competition ED is a solution-based assay, competition ELISA depends on interactions at the solid-liquid interface (Fig. 5). In contrast to the competition ED-UPLC/MS/MS method, the IC_50_ values calculated from competition ELISA were in the micromolar range, which suggest low affinity antibodies (Table 3 and ESM Fig. S11). Collectively, the IC_50_ values derived from competition ELISA were 10^3^–10^4^ higher than the *K*_d_ from competition ED-UPLC/MS/MS. However, we believe that these IC_50_ values are not true reflections of drug-antibody interactions. In competition ELISA, hapten-antibody interactions occur at the solid-liquid interface and, thus, are prone to avidity effects. It could be envisioned that the two arms of the IgG antibodies were tightly engaged to the haptens of the coating antigen; thus, higher concentrations of the competing drugs are needed to disrupt the hapten-antibody interactions. Besides underestimation of antibody affinity (i.e., high IC_50_), another limitation associated with competition ELISA is ambiguity in the interpretation of results. In the case of anti-DiAmHap, there are at least two interpretations for IC_50_ >10^3^ μM. As we originally proposed for anti-DiAmHap and anti-DiPrOxyHap, either the hapten antibodies simply do not cross-react with the competing drugs, or the affinity of the hapten antibodies to the original hapten design is extremely high [25, 30]. The latter interpretation is consistent with our previous findings [30] and the findings of Pravetoni et al., which demonstrated the higher affinity of hapten antibodies to haptenic surrogate than the target drugs [15]. In addition, the avidity effects associated with competition ELISA further increase the interactions between the haptenic surrogate and the hapten antibodies. This ambiguity can be resolved by considering the *b* values derived from competition ED-UPLC/MS/MS (ESM Tables S1–S3). Extending those findings, the IC_50_ values of anti-DiAmHap and anti-DiPrOxyHap to 6-AM and morphine are simply due to weak drug-antibody interactions.

Although there was an underestimation of binding affinities (i.e., high IC_50_) with competition ELISA [14], it is noteworthy to mention that both methods gave similar trends, i.e., polyclonal antibody with high binding affinities for heroin, 6-AM, and morphine was consistently predicted by both methods. In light of the results, we have observed recurring themes that could serve as guiding principles in the design of a heroin vaccine (Fig. 1b). First, when linker is at the C3 position, the induced antibodies (anti-6-AcMorHap and anti-6-PrOxyHap) cross-reacted with heroin, 6-AM, and morphine. Second, when linker is at the C6 position, the induced antibodies (anti-MorHap) cross-reacted with both 6-AM and morphine, but poorly with heroin. Third, when the linker is at the bridge nitrogen, the induced antibodies (anti-DiAmHap and anti-DiPrOxyHap) had low cross-reactivity to both 6-AM and morphine. Heroin contains two acetyl groups at the C3 and C6 position, while DiAmHap and DiPrOxyHap have acetamide and propan-2-one groups, respectively. The binding affinity data suggest that modifications at this position dramatically affect the cross-reactivity of the induced antibodies to heroin. These results are in agreement with previous reports that hydrolytically stable N-linked haptens tended to generate antibodies that were highly specific to the parent hapten and had no cross-reactivity to other drugs [49–52]. It has been postulated that antibody titer and antibody affinity are crucial for the efficacy of drug of abuse vaccines [1, 2]. Previously, we demonstrated that mice immunized with TT-DiAmHap and TT-DiPrOxyHap showed partial protection and no protection against heroin challenge, respectively [25, 30]. In terms of binding affinity, anti-DiAmHap is superior to anti-DiPrOxyHap (Fig. 4b and ESM Table S3). The protection against heroin challenge could be attributed to the presence of high titer, but low affinity antibodies to heroin and 6-AM. Based on IC_50_ and antibody titer from ELISA and *K*_d_ and % heroin bound from competition ED-UPLC/MS/MS, it is clear that 6-AcMorHap and 6-PrOxyHap are the lead hapten designs.

Despite the ability of competition ED-UPLC/MS/MS to measure *K*_d_, the assay system does have some limitations. Due to the use of dilute sera, the method cannot measure the affinity of polyclonal antibodies that poorly bind 6-AM and morphine. Within the context of a heroin vaccine, it can be argued that the determination of antibodies with high *K*_d_ values (>1 μM, low affinity antibodies) is unimportant, since these antibodies are expected to bind low amounts of heroin and its metabolites and, consequently, have little role in efficacy. The competition ED-UPLC/MS/MS also cannot be used for drugs that have a significant binding to plasma proteins even at high sera dilutions. Overall, competition ED-UPLC/MS/MS is a simple method for determining the binding affinities of antibodies. It is a convenient method because it does not involve the use of radioactive tracers, and all the reagents for the assay are commercially available. This nonradioactive method only requires 5–10 μL of anti-hapten sera to generate a binding curve and has the potential applications for studying the binding affinities of antibodies induced by small molecule hapten-based immunogens. In addition, competition ED-UPLC/MS/MS resolved the ambiguity of the IC_50_ values derived from competition ELISA.

## Electronic supplementary material

ESM 1 (PDF 1.90 mb)

## References

[1] Kosten TR, Domingo CB (2013). Can you vaccinate against substance abuse?. Expert Opin Biol Ther.

[2] Janda KD, Treweek JB (2012). Vaccines targeting drugs of abuse: is the glass half-empty or half-full?. Nat Rev Immunol.

[3] Rook EJ, van Ree JM, van den Brink W, Hillebrand MJ, Huitema AD, Hendriks VM, Beijnen JH (2006). Pharmacokinetics and pharmacodynamics of high doses of pharmaceutically prepared heroin, by intravenous or by inhalation route in opioid-dependent patients. Basic Clin Pharmacol Toxicol.

[4] Stowe GN, Schlosburg JE, Vendruscolo LF, Edwards S, Misra KK, Schulteis G, Zakhari JS, Koob GF, Janda KD (2011). Developing a vaccine against multiple psychoactive targets: a case study of heroin. CNS Neurol Disord: Drug Targets.

[5] Bogen IL, Boix F, Nerem E, Morland J, Andersen JM (2014). A monoclonal antibody specific for 6-monoacetylmorphine reduces acute heroin effects in mice. J Pharmacol Exp Ther.

[6] Carroll FI, Blough BE, Pidaparthi RR, Abraham P, Gong PK, Deng L, Huang X, Gunnell M, Lay JO, Peterson EC, Owens SM (2011). Synthesis of mercapto-(+)-methamphetamine haptens and their use for obtaining improved epitope density on (+)-methamphetamine conjugate vaccines. J Med Chem.

[7] Jalah R, Torres OB, Mayorov AV, Li F, Antoline JF, Jacobson AE, Rice KC, Deschamps JR, Beck Z, Alving CR, Matyas GR (2015). Efficacy, but not antibody titer or affinity, of a heroin hapten conjugate vaccine correlates with increasing hapten densities on tetanus toxoid, but not on CRM197 carriers. Bioconjug Chem.

[8] Engvall E, Perlmann P (1971). Enzyme-linked immunosorbent assay (ELISA). Quantitative assay of immunoglobulin G. Immunochemistry.

[9] Van Weemen BK, Schuurs AH (1971). Immunoassay using antigen-enzyme conjugates. FEBS Lett.

[10] Engvall E, Perlmann P (1972). Enzyme-linked immunosorbent assay, ELISA. 3. Quantitation of specific antibodies by enzyme-labeled anti-immunoglobulin in antigen-coated tubes. J Immunol.

[11] Plested JS, Coull PA, Gidney MA (2003). ELISA. Methods Mol Med.

[12] Devey ME, Bleasdale K, Lee S, Rath S (1988). Determination of the functional affinity of IgG1 and IgG4 antibodies to tetanus toxoid by isotype-specific solid-phase assays. J Immunol Methods.

[13] Rath S, Stanley CM, Steward MW (1988). An inhibition enzyme immunoassay for estimating relative antibody affinity and affinity heterogeneity. J Immunol Methods.

[14] Bremer PT, Schlosburg JE, Lively JM, Janda KD (2014). Injection route and TLR9 agonist addition significantly impact heroin vaccine efficacy. Mol Pharmaceutics.

[15] Pravetoni M, Keyler DE, Pidaparthi RR, Carroll FI, Runyon SP, Murtaugh MP, Earley CA, Pentel PR (2012). Structurally distinct nicotine immunogens elicit antibodies with non-overlapping specificities. Biochem Pharmacol.

[16] Way EL, Kemp JW, Young JM, Grassetti DR (1960). The pharmacologic effects of heroin in relationship to its rate of biotransformation. J Pharmacol Exp Ther.

[17] Lockridge O, Mottershaw-Jackson N, Eckerson HW, La Du BN (1980). Hydrolysis of diacetylmorphine (heroin) by human serum cholinesterase. J Pharmacol Exp Ther.

[18] Owen JA, Nakatsu K (1983). Diacetylmorphine (heroin) hydrolases in human blood. Can J Physiol Pharmacol.

[19] Salmon AY, Goren Z, Avissar Y, Soreq H (1999). Human erythrocyte but not brain acetylcholinesterase hydrolyses heroin to morphine. Clin Exp Pharmacol Physiol.

[20] Redinbo MR, Bencharit S, Potter PM (2003). Human carboxylesterase 1: from drug metabolism to drug discovery. Biochem Soc Trans.

[21] Torres OB, Jalah R, Rice KC, Li F, Antoline JF, Iyer MR, Jacobson AE, Boutaghou MN, Alving CR, Matyas GR (2014). Characterization and optimization of heroin hapten-BSA conjugates: method development for the synthesis of reproducible hapten-based vaccines. Anal Bioanal Chem.

[22] Wu G (2010). Assay development: fundamentals and practices.

[23] Müller R (1983). Determination of affinity and specificity of anti-hapten antibodies by competitive radioimmunoassay. Methods Enzymol.

[24] Pichini S, Altieri I, Pellegrini M, Zuccaro P, Pacifici R (1999). The role of liquid chromatography-mass spectrometry in the determination of heroin and related opioids in biological fluids. Mass Spectrom Rev.

[25] Li F, Cheng K, Antoline JF, Iyer MR, Matyas GR, Torres OB, Jalah R, Beck Z, Alving CR, Parrish DA, Deschamps JR, Jacobson AE, Rice KC (2014). Synthesis and immunological effects of heroin vaccines. Org Biomol Chem.

[26] Droupadi PR, Meyers EA, Linthicum DS (1994). Spectroscopic evidence for charge-transfer complexation in monoclonal antibodies that bind opiates. J Protein Chem.

[27] Gottas A, Oiestad EL, Boix F, Ripel A, Thaulow CH, Pettersen BS, Vindenes V, Morland J (2012). Simultaneous measurement of heroin and its metabolites in brain extracellular fluid by microdialysis and ultra performance liquid chromatography tandem mass spectrometry. J Pharmacol Toxicol Methods.

[28] Fahnestock ML, Johnson JL, Feldman RM, Tsomides TJ, Mayer J, Narhi LO, Bjorkman PJ (1994). Effects of peptide length and composition on binding to an empty class I MHC heterodimer. Biochemistry.

[29] Matyas GR, Mayorov AV, Rice KC, Jacobson AE, Cheng K, Iyer MR, Li F, Beck Z, Janda KD, Alving CR (2013). Liposomes containing monophosphoryl lipid A: a potent adjuvant system for inducing antibodies to heroin hapten analogs. Vaccine.

[30] Matyas GR, Rice KC, Cheng K, Li F, Antoline JF, Iyer MR, Jacobson AE, Mayorov AV, Beck Z, Torres OB, Alving CR (2014). Facial recognition of heroin vaccine opiates: type 1 cross-reactivities of antibodies induced by hydrolytically stable haptenic surrogates of heroin, 6-acetylmorphine, and morphine. Vaccine.

[31] Csuk R, Vasileva G, Barthel A (2012). Towards an efficient preparation of hydromorphone. Synthesis.

[32] Uemura S, Toshimitsu A, Okano M (1978). Nitration of aromatic hydrocarbons and ipso-nitrosodemetallation of arylmetal compounds in sodium nitrite–trifluoroacetic acid. J Chem Soc Perkin Trans.

[33] Galibert M, Renaudet O, Dumy P, Boturyn D (2011). Access to biomolecular assemblies through one-pot triple orthogonal chemoselective ligations. Angew Chem Int Ed Engl.

[34] Information on anti-morphine antibody (ab1060). http://www.abcam.com/morphine-antibody-ab1060.html. Accessed on 23 June 2015

[35] Alving CR, Matyas GR, Torres O, Jalah R, Beck Z (2014). Adjuvants for vaccines to drugs of abuse and addiction. Vaccine.

[36] Smith PT, Hirst M, Gowdey CW (1978). Spontaneous hydrolysis of heroin in buffered solution. Can J Physiol Pharmacol.

[37] Landsteiner K, Jacobs J (1935). Studies on the sensitization of animals with simple chemical compounds. J Exp Med.

[38] Landsteiner K, Jacobs J (1936). Studies on the sensitization of animals with simple chemical compounds II. J Exp Med.

[39] Stowe GN, Vendruscolo LF, Edwards S, Schlosburg JE, Misra KK, Schulteis G, Mayorov AV, Zakhari JS, Koob GF, Janda KD (2011). A vaccine strategy that induces protective immunity against heroin. J Med Chem.

[40] Pryde DC, Jones LH, Gervais DP, Stead DR, Blakemore DC, Selby MD, Brown AD, Coe JW, Badland M, Beal DM, Glen R, Wharton Y, Miller GJ, White P, Zhang N, Benoit M, Robertson K, Merson JR, Davis HL, McCluskie MJ (2013). Selection of a novel anti-nicotine vaccine: influence of antigen design on antibody function in mice. PLoS One.

[41] Spector S, Parker CW (1970). Morphine: radioimmunoassay. Science.

[42] Koida M, Takahashi M, Kaneto H (1974). The morphine 3-glucuronide directed antibody: its immunological specificity and possible use for radioimmunoassay of morphine in urine. Jpn J Pharmacol.

[43] Hoskin PJ, Hanks GW (1990). Morphine: pharmacokinetics and clinical practice. Br J Cancer.

[44] Zhang F, Xue J, Shao J, Jia L (2012). Compilation of 222 drugs’ plasma protein binding data and guidance for study designs. Drug Discov Today.

[45] Hardman JG, Limbird LE, Gilman AG (2001). Goodman & Gilman’s the pharmacological basis of therapeutics.

[46] Li B, Stribley JA, Ticu A, Xie W, Schopfer LM, Hammond P, Brimijoin S, Hinrichs SH, Lockridge O (2000). Abundant tissue butyrylcholinesterase and its possible function in the acetylcholinesterase knockout mouse. J Neurochem.

[47] Eng H, Niosi M, McDonald TS, Wolford A, Chen Y, Simila ST, Bauman JN, Warmus J, Kalgutkar AS (2010). Utility of the carboxylesterase inhibitor bis-para-nitrophenylphosphate (BNPP) in the plasma unbound fraction determination for a hydrolytically unstable amide derivative and agonist of the TGR5 receptor. Xenobiotica.

[48] Hatfield MJ, Potter PM (2011). Carboxylesterase inhibitors. Expert Opin Ther Pat.

[49] Morris BA, Robinson JD, Piall E, Aherne GW, Marks V (1975). Proceedings: development of a radioimmunoassay for morphine having minimal cross-reactivity with codeine. J Endocrinol.

[50] Findlay JW, Butz RF, Jones EC (1981). Relationships between immunogen structure and antisera specificity in the narcotic alkaloid series. Clin Chem.

[51] Beike J, Blaschke G, Mertz A, Kohler H, Brinkmann B (1999). A specific immunoassay for the determination of morphine and its glucuronides in human blood. Int J Legal Med.

[52] Usagawa T, Itoh Y, Hifumi E, Takeyasu A, Nakahara Y, Uda T (1993). Characterization of morphine-specific monoclonal antibodies showing minimal cross-reactivity with codeine. J Immunol Methods.

