# Supplementary material for: A simple nonradioactive method for the determination of the binding affinities of antibodies induced by hapten bioconjugates for drugs of abuse
Source: Anal Bioanal Chem. 2015 Dec 16;408:1191–204. doi: 10.1007/s00216-015-9223-z (PMC4718952; doi:10.1007/s00216-015-9223-z)
Supplement: Supplementary file 1 — (PDF 1.90 mb) [file 216_2015_9223_MOESM1_ESM.pdf]

Analytical and Bioanalytical Chemistry

Electronic Supplementary Material

A simple nonradioactive method for the determination of the binding affinities of antibodies induced by hapten bioconjugates for drugs of abuse

Oscar B. Torres, Joshua F. G. Antoline, Fuying Li, Rashmi Jalah, Arthur E. Jacobson, Kenner C. Rice, Carl R. Alving, Gary R. Matyas

Contents

INDEX	PAGE
Analytical Characterization of **6**, **7**, **9** and 6-AcMorHap	S3-S6
Preparation of anti-hapten sera	S7
Strategy of Conventional ED and Competition ED for anti-hapten sera	S7
ELISA and competition ELISA	S8-S9
Synthesis BSA-hapten ELISA coating agent	S9-10
**Table S1** Affinity of anti-hapten sera to 6-AM at different sera dilutions	S11
**Table S2** Affinity of anti-hapten sera to morphine at different sera dilutions	S11
**Table S3** *b* values of anti-DiAmHap and anti-DiPrOxyHap to 6-AM at low sera dilutions	S12
**Table S4** UPLC Gradient Profile	S12
**Fig. S1** D_3-tracers that were used in competition ED-UPLC/MS/MS	S13
**Fig. S2** ^{1}H-NMR of 6-AcMorHap	S13
**Fig. S3** ^{13}C-NMR of 6-AcMorHap	S14
**Fig. S4** Competitive inhibition in solution-based ED of 6-AM and morphine to the binding of anti-hapten sera or monoclonal morphine antibody (ab1060) to D_3-tracer (D_3-6-AM or D_3-morphine)	S14
**Fig. S5** LC MS spectrum of D_3-heroin	S15
**Fig. S6** LC MS spectrum of heroin	S15
**Fig. S7** LC MS spectrum of D_3-6-AM	S16
**Fig. S8** LC MS spectrum of 6-AM	S16
**Fig. S9** LC MS spectrum of D_3-morphine	S17
**Fig. S10** LC MS spectrum of morphine	S17
**Fig. S11** Competitive inhibition of heroin and its degradation products to the binding of anti-hapten sera to BSA-hapten coated ELISA plates	S18
**Fig. S12** Synthesis of hapten-bioconjugate (Protein= TT) and ELISA coating agent (Protein= BSA) using maleimide-thiol chemistry	S18

Analytical Characterization of 6, 7, 9 and 6-AcMorHap

ACS reagent grade ethanol and anhydrous (DriSolv) toluene were purchased from EMD Millipore *via* VWR International (Suwanee, GA, USA). N,N'-Dicyclohexylcarbodiimide (DCC, ≥ 99%), 4-(Dimethylamino)pyridine (DMAP, ≥ 99%), tetrahydrofuran (THF, ≥ 99.9%), ACS reagent grade chemical reagents and solvents that were used in organic synthesis were purchased from Sigma-Aldrich (Saint Louis, MO, USA). All melting points were determined on a Thomas-Hoover melting-point apparatus and are uncorrected. Proton nuclear magnetic resonance (^{1}H NMR, 500 MHz) and carbon nuclear magnetic resonance (^{13}C NMR, 100 MHz) spectra were recorded on a Bruker 500 instrument in $CDCl_3$ (unless otherwise noted) with the values given in ppm (TMS as internal standard) and J (Hz) assignments of ^{1}H resonance coupling. The high resolution mass spectra (HRMS) were recorded using electrospray ionization (ESI) on a Waters LCT Premier time-of-flight (TOF) mass spectrometer. Thin-layer chromatography (TLC) was performed on 0.25 mm Analtech GHLF silica gel. Flash column chromatography was performed with Bodman silica gel LC 60 A. Units for $[\alpha]_D$ values were given in 10^{-1} deg cm^2 g^{-1}.

(7S,7aR)-11-Chloro-3-methyl-9-nitro-2,3,4,4a,5,6,7,7a-octahydro-1H-4,12-methanobenzofuro[3,2-e]isoquinolin-7-ol **(6, 1-chloro-3-nitro-dihydromorphine)**

To a stirred solution of 1-chloro-3-nitro-dihydromorphinone (**5**, 178 mg, 0.51 mmol) in anhydrous THF (12 mL) at 0 ^oC was added K-selectride (0.50 mL, 1.0 M in THF) dropwise. The resulting solution was stirred for 3 h at 0 ^oC. THF was removed in vacuo and the residue was purified by silica gel column chromatography (eluent: $CHCl_3$:

MeOH : NH_4OH = 90 : 10: 1) to give 1-chloro-3-nitro-dihydromorphine (**6**, 148 mg, 83% yield) as a foam.

$[\alpha]_D^{20}$ = -7.8° (*c* 1.0, $CHCl_3$); ^{1}H NMR ($CDC1_3$) δ: 7.90 (s, 1H), 4.86 (d, 1H, *J* = 5.5 Hz), 4.18-4.23 (m, 1H), 3.19 (t, 1H, *J* = 2.5 Hz), 3.05 (d, 1H, *J* = 20.0 Hz), 2.55 (dd, 1H, *J* = 12.0, 4.0 Hz), 2.40 (s, 3H), 2.34 (dd, 1H, *J* = 20.0, 5.5 Hz), 2.27 (ddd, 1H, *J* = 13.0, 5.0, 2.5 Hz), 2.17 (s-br, 1H), 2.05 (dt, 1H, *J* = 12.0, 3.5 Hz), 1.93 (dt, 1H, *J* = 12.0, 5.0 Hz), 1.65-1.83 (m, 3H), 1.45-1.54 (m, 1H), 1.36-1.43 (m, 1H), 1.13 (ddd, 1H, *J* = 20.0, 12.5, 3.5 Hz); ^{13}C NMR ($CDCl_3$) δ: 152.83, 139.92, 135.35, 130.44, 124.26, 123.22, 93.09, 77.37, 66.48, 58.89, 46.12, 42.96, 41.78, 36.54, 28.14, 20.51, 18.30 ppm; HRMS (EI) calcd for $C_{17}H_{20}ClN_2O_4$ (M + H^+): 351.1112, found: 351.1105.

(7S,7aR)-9-Amino-3-methyl-2,3,4,4a,5,6,7,7a-octahydro-1H-4,12-methanobenzofuro[3,2-e]isoquinolin-7-ol **(7, 3-amino-dihydromorphine)**

10% (w/w) Pd/C (446 mg) was added to a solution of **6** (140 mg, 0.40 mmol) in 20% aqueous acetic acid (50 mL) and the mixture was stirred under H_2 (45 psi) overnight at room temperature. Then, the reaction mixture was filtered through a Celite pad, and concentrated under reduced pressure to afford the crude product. This was purified through silica gel column chromatography (eluent: $CHCl_3$: MeOH : NH_4OH = 90 : 10: 1) to give amine 3- amino-dihydromorphine (**7**, 82 mg, 72%).

$[\alpha]_D^{20}$ = -96.0° (*c* 0.60, $CHCl_3$); ^{1}H-NMR (CD_3OD) δ: 6.58 (d, 1H, *J* = 8.0 Hz), 6.54 (d, 1H, *J* = 8.0 Hz), 4.56 (d, 1H, *J* = 5.0 Hz), 4.04 (s-br, 1H), 3.67 (s-br, 1H), 3.58 (s-br, 2H), 3.16 (d, 1H, *J* = 12.0 Hz), 2.80 (s, 3H), 2.60-3.0 (m, 3H), 2.27-2.40 (m, 1H), 1.78 (dd, 1H, *J* = 13.5, 3.0 Hz), 1.54-1.70 (m, 3H), 1.53-1.65 (m, 1H), 1.45-1.52 (m, 1H),

1.10-1.25 (m, 1H); ^{13}C NMR ($CDCl_3$) δ: 146.80, 129.60, 128.00, 124.91, 119.42, 118.35, 89.28, 77.34, 66.15, 62.37, 46.52, 42.67, 41.30, 35.41, 29.71, 20.81, 17.30 ppm; HRMS (EI) calcd for $C_{17}H_{23}N_2O_2$ (M + H^+): 287.1760, found: 287.1755.

N-((7*S*,7a*R*)-7-hydroxy-3-methyl-2,3,4,4a,5,6,7,7a-octahydro-1*H*-4,12-methanobenzofuro[3,2-*e*]isoquinolin-9-yl)-3-(tritylthio)propanamide **(9, 3-tritylthiopropanamide-dihydromorphine)**

Tritylmercaptopropionic acid NHS-ester (**8**) was prepared from the reaction of N-hydroxysuccinimide and tritylmercaptopropionic acid with DCC as previously reported by Li *et al*. Triethylamine (50 mg, 0.49 mmol) was added to a stirred mixture of **7** (70 mg, 0.244 mmol), **8** (142 mg, 0.318 mmol), and DMAP (10 mg) in anhydrous methylene chloride (6 mL) at 0 ^{o}C. The resulting solution was warmed to 30 ^{o}C and stirred for 24 h. The solution was concentrated in vacuo and the residue was purified by silica gel column chromatography (eluent: $CHCl_3$: MeOH : NH_4OH = 90 : 10: 1) to give the solid trityl compound 3-tritylthiopropanamide-dihydromorphine (**9**, 134 mg, 89% yield).

$[\alpha]_D^{20}$ = -41.2° (*c* 1.0, $CHCl_3$); ^{1}H NMR (CDC1$_3$) δ: 7.46 (d, 6H, *J* = 7.5 Hz), 7.21-7.34 (m, 9H), 7.03 (s-br, 1H), 6.90 (d, 1H, *J* = 8.0 Hz), 6.63 (d, 1H, *J* = 8.0 Hz), 4.56 (d, 1H, *J* = 5.2 Hz), 4.11-4.17 (m, 1H), 3.08-3.14 (m, 1H), 3.02 (d, 1H, *J* = 18.4 Hz), 2.62 (t, 2H, *J* = 7.2 Hz), 2.55 (dd, 1H, *J* = 12.0, 4.0 Hz), 2.42 (s, 3H), 2.41 (dd, 1H, *J* = 18.4, 5.6 Hz), 2.18-2.34 (m, 3H), 2.11 (dd, 1H, *J* = 15.2, 7.6 Hz), 2.04 (d, 1H, *J* = 20.4 Hz), 1.87 (dt, 1H, *J* = 12.0, 4.8 Hz), 1.75-1.83 (m, 1H), 1.65 (dd, 1H, *J* = 12.4, 2.0 Hz), 1.35-1.45 (m, 1H), 1.25-1.34 (m, 2H); ^{13}C NMR ($CDCl_3$) δ: 169.49, 151.65, 144.62, 132.32, 130.76, 129.61, 128.01, 126.76, 123.93, 118.83, 116.35, 91.80, 66.94, 66.80,

59.71, 47.01, 42.90, 42.38, 41.32, 36.54, 35.75, 28.21, 27.60, 20.57, 18.42 ppm; HRMS (EI) calcd for $C_{39}H_{41}N_2O_3S$ (M + H^+): 617.2838, found: 617.2817.

(7S,7aR)-3-methyl-9-(3-(tritylthio)propanamido)-2,3,4,4a,5,6,7,7a-octahydro-1H-4,12-methanobenzofuro[3,2-e]isoquinolin-7-yl acetate **(6-AcMorHap)**

To a stirred mixture of **9** (80 mg, 0.13 mmol), triethylamine (39 mg, 0.39 mmol) and DMAP (10 mg) in anhydrous methylene chloride (5 mL) at 0 ^oC was added acetic anhydride (27 mg, 0.26 mmol). The resulting solution was slowly warmed to room temperature and stirred overnight. The solution was concentrated in vacuo and the residue was purified by silica gel column chromatography (eluent: $CHCl_3$: MeOH : NH_4OH = 90 : 10: 1) to give 6-AcMorHap as a yellow solid (134 mg, 89% yield). The solid was lyophilized with *tert*-butanol to give a yellow powder, 6-AcMorHap.

$[\alpha]_D^{20}$ = -47.3° (*c* 2.0, $CHCl_3$); ^1H NMR ($CDC1_3$) δ: 7.92 (d, 1H, *J* = 8.4 Hz), 7.47 (d, 6H, *J* = 7.6 Hz), 7.32 (t, 6H, *J* = 7.2 Hz), 7.20-7.25 (m, 3H), 7.03 (s-br, 1H), 6.66 (d, 1H, *J* = 8.4 Hz), 5.30 (m, 1H), 4.68 (d, 1H, *J* = 5.2 Hz), 3.11 (m, 1H), 3.03 (d, 1H, *J* = 18.4 Hz), 2.58-2.70 (m, 2H), 2.52 (dd, 1H, *J* = 12.0, 4.0 Hz), 2.41 (s, 3H), 2.40 (dd, 1H, *J* = 18.4, 5.6 Hz), 2.18-2.30 (m, 2H), 2.00-2.10 (m, 3H), 1.88 (dt, 1H, *J* = 12.0, 4.8 Hz), 1.68 (s, 3H), 1.60-1.70 (m, 2H), 1.40-1.53 (m, 2H), 1.12-1.20 (m, 1H); ^{13}C NMR ($CDCl_3$) δ: 170.21, 169.01, 147.57, 144.70, 129.98, 129.65, 127.99, 126.74, 120.18, 119.06, 118.81, 88.32, 77.25, 68.15, 66.93, 59.57, 46.97, 42.95, 42.34, 41.58, 36.66, 36.60, 27.69, 25.79, 20.71, 20.29, 19.08 ppm; HRMS (EI) calcd for $C_{41}H_{43}N_2O_4S$ (M + H^+): 659.2944, found: 659.2938.

Preparation of anti-hapten sera

Heroin haptens 6-AcMorHap, 6-PrOxyHap, MorHap, DiAmHap and DiPrOxyHap were coupled to tetanus toxoid (TT) and mixed with liposomes containing monophosphoryl lipid A as adjuvant (Fig. S12). Adjuvants for vaccines to drugs of abuse and addiction were reviewed by Alving *et al*. Vaccine formulation and immunization (2 doses at weeks 0 and 6) were previously described by Li *et al*. and Matyas *et al*. Week 9 sera (3 weeks after two immunizations) from the 5 individual mice were pooled to yield the test samples (anti-hapten sera) for competition ED-UPLC/MS/MS and competition ELISA.

Strategy of Conventional ED and Competition ED for anti-hapten sera

In a conventional ED, polyclonal sera (sample chamber) are allowed to equilibrate with different drug concentrations (buffer chamber). At equilibrium, the concentrations of the free drug, $[\text{drug}]_{free}$, and bound drug to the antibody, $[\text{drug}]_{bound}$, can be calculated. The K_d is derived from the plot of binding coefficient (B) versus $[\text{drug}]_{free}$, where B is $[\text{drug}]_{bound}/[\text{Antibody}]_{total}$. Since the determination of total antibody concentration in polyclonal sera is challenging, the use of conventional equilibrium dialysis to determine the affinity of hapten antibodies is not practical.

In competition ED, polyclonal sera are mixed with fixed concentration of radiolabeled tracer (sample chamber) and are allowed to equilibrate with different concentrations of the competitive inhibitor (buffer chamber). In Müller's method, the concentration of the antibody is not required to calculate K_d. Herein, a modified version of Müller's method with D_3-labeled drug as a tracer and the corresponding unlabeled drug as competitive inhibitor was employed to calculate K_d.

ELISA and competition ELISA

Bovine Serum Albumin (BSA) that was used as blocking reagent for ELISA, Tris.HCl and NaCl, used for the preparation of ELISA blocker (1% BSA in 20 mM Tris-0.15 M sodium chloride, pH 7.4), Tween 20®, used to prepare ELISA wash buffer (20 mM Tris-0.15 M sodium chloride-0.05% Tween 20®), and morphine sulfate (≥ 98%) were purchased from Sigma-Aldrich (Saint Louis, MO, USA). The 2,2'-Azino-di(3-ethylbenzthiazoline-6-sulfonate) (ABTS) peroxidase substrate system was purchased from KPL, Inc. (Gaithersburg, MD, USA). Ultrapure 10 % sodium dodecyl sulfate (SDS) was purchased from Thermo Fisher Scientific (Rockville, MD, USA). Heroin.HCl.H_2O and 6-AM.HCl powders were Certified Reference Materials and were purchased from Lipomed.

The synthesis of BSA-hapten was described *vide infra*. BSA-hapten (100 μL, 1 μg/mL BSA in DPBS) was added to the ELISA plates and incubated at 4 °C overnight. The excess BSA-hapten was removed and the remainder of the ELISA was conducted as described previously by Jalah *et al*. Briefly, the plates were incubated with blocker for 2 h. Mouse sera were serially diluted in blocker and added to the plates in triplicates. Following incubation for 2 h at RT, the plates were washed with wash buffer. Peroxidase linked-sheep anti-mouse IgG diluted in blocker (1:1000) was added and the plates were incubated for 1 h at RT. The plates were washed and ABTS peroxidase substrate system (100 μL/well) was added. After incubation at RT for 1 h, 1 % SDS (100 μL/well) was added to stop the reaction and the absorbance was read at 405 nm.

Pooled sera from the 5 individual mice were diluted in blocker to give an ELISA absorbance of approximately 1.5. Heroin.HCl, 6-AM.HCl and morphine sulfate were

dissolved in blocker. The inhibitors were diluted in 10-fold increments in a 96 well plate and mixed with diluted sera to give the final inhibitor concentrations between 0 to 10^3 μM for all opiates. Each serum-inhibitor mixture was incubated for 30 min at RT and added to ELISA plates that were coated with BSA-hapten conjugates. The ELISA was processed as described above. Normalized curves obtained from competition ELISAs were used to calculate the 50% inhibition concentration (IC_{50}) values, which is the concentration of the drugs required to inhibit 50% of the binding of anti-hapten antibodies to surface immobilized BSA-hapten conjugates.

Synthesis BSA-hapten ELISA coating agent

BSA, used for synthesizing the BSA-hapten ELISA coating agents, NHS-$(PEG)_2$-maleimide linker (SM-$(PEG)_2$), spin desalting column (Zeba, 7K molecular weight cut-off (MWCO)), dialysis cassettes (Slide-A-Lyzer G2, 10K MWCO), bicinchoninic acid (BCA) protein assay kit, and phosphate buffered saline (PBS, 100 mM sodium phosphate, 150 mM NaCl, pH 7.2) that was used for the coupling reaction were purchased from Thermo Fisher Scientific (Rockville, MD, USA). Dimethylsulfoxide (DMSO) was purchased from Sigma-Aldrich (Saint Louis, MO). Dulbecco's phosphate buffered saline (DPBS, 10 mM Na_2HPO_4, 1.8 mM KH_2PO_4, 2.7 mM KCl, 137 mM NaCl, pH 7.4) that was used for dialysis of BSA-hapten conjugates was purchased from Quality Biological Inc. (Gaithersburg, MD, USA).

Haptens were coupled to BSA using the optimized bioconjugation method described by Torres *et al* (Fig. S12). An aliquot of BSA solution (250 μL, 10 mg/mL in PBS) is added to 750 μL of PBS and subsequently treated with SM-$(PEG)_2$ linker (1.5

μL, 250 mM in DMSO). The reaction mixture was incubated for 2 h at RT. The molar conjugation ratio of the linker to BSA was 10. Excess linker was removed by spin desalting column to yield the BSA-maleimide intermediate.

An aqueous solution of the hapten was prepared as described by Torres *et al*. The deprotected hapten was added dropwise to BSA-maleimide and the reaction mixture was incubated at RT for 2 h. Excess hapten was removed by overnight dialysis in DPBS at 4 °C. The BSA-hapten conjugate was membrane filtered and the protein concentration was determined by BCA assay. The hapten density of all the BSA-hapten conjugates was ~5 as judged by matrix assisted laser desorption ionization time-of-flight mass spectrometry (MALDI-TOF MS).

Table S1 Affinity of anti-hapten sera to 6-AM at different sera dilutions

Anti-hapten sera	Sera dilution					
	1:400		1:800		1:1600	
	b[a]	K_d, nM[b]	*b*[a]	K_d, nM[b]	*b*[a]	K_d, nM[b]
6-AcMorHap	0.87 ± 0.002	0.28 ± 0.02[c]	0.69 ± 0.015	0.56 ± 0.05	0.54 ± 0.016	0.62 ± 0.16
6-PrOxyHap	0.75 ± 0.009	0.93 ± 0.09	0.50 ± 0.023	1.20 ± 0.19	0.40 ± 0.017	1.11 ± 0.13
MorHap	0.43 ± 0.004	2.94 ± 0.34	0.29 ± 0.052	---	0.17 ± 0.012	---
DiAmHap	0.07 ± 0.0001	---	0	---	0	---
DiPrOxyHap	0	---	0	---	0	---

[a] Fraction of D_3-6-AM bound to the polyclonal antibody in the absence of 6-AM. The K_d values were not determined for assay conditions with *b* values of < 0.4 (dash line).

[b] All K_d values were calculated using Müller's equation and are the mean of triplicate determinations ± standard deviation.

[c] The K_d value at 1:400 dilution is different to the K_d derived from 1:800 ($p < 0.05$) and 1:1600 ($p < 0.05$, multiple comparison using one-way ANOVA).

Table S2 Affinity of anti-hapten sera to morphine at different sera dilutions

Anti-hapten sera	Sera dilution					
	1:400		1:800		1:1600	
	b[a]	K_d, nM[b]	*b*[a]	K_d, nM[b]	*b*[a]	K_d, nM[b]
6-AcMorHap	0.87 ± 0.005	0.30 ± 0.01[c]	0.68 ± 0.004	0.55 ± 0.08	0.52 ± 0.035	0.46 ± 0.04
6-PrOxyHap	0.59 ± 0.029	1.55 ± 0.05	0.40 ± 0.023	1.40 ± 0.16	0.30 ± 0.009	0[d]
MorHap	0.50 ± 0.022	1.88 ± 0.46	0.30 ± 0.021	---	0.18 ± 0.010	---
DiAmHap	0	---	0	---	0	---
DiPrOxyHap	0	---	0	---	0	---

[a] Fraction of D_3-morphine bound to the polyclonal antibody in the absence of morphine.

[b] All K_d values were calculated using Müller's equation and are the mean of triplicate determinations ± standard deviation.

[c] The K_d value at 1:400 dilution is different to the K_d derived from 1:800 ($p < 0.01$) and 1:1600 ($p < 0.05$, multiple comparison using one-way ANOVA).

[d] The K_d value at 1:1600 dilution is negative and is different to the K_d derived from 1:400 ($p < 0.001$) and 1:800 ($p < 0.001$, multiple comparison using one-way ANOVA).

Table S3 *b* values of anti-DiAmHap and anti-DiPrOxyHap to 6-AM at low sera dilutions

Anti-hapten sera	Sera dilution[a]			
	1:25	1:50	1:100	1:200
DiAmHap	0.62 ± 0.02	0.48 ± 0.02	0.28 ± 0.02	0.13 ± 0.11
DiPrOxyHap	0.09 ± 0.02	0.05 ± 0.04	0	0

[a] *b* values are the mean of triplicate determinations ± standard deviation. The *b* values for morphine at all dilutions are ~0.

Table S4 UPLC Gradient Profile

Time (min)	**A**: 10 mM NH_4HCOO pH 3.1 (%)	**B**: MeOH (%)
0	100	0
0.50	100	0
2.70	90	10
3.30	80	20
4.60	20	80
4.61	0	100
5.20	0	100
5.21	100	0
8.00	100	0

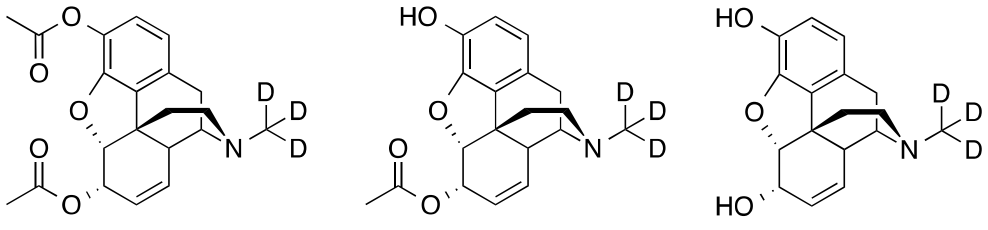

D3-heroin D3-6-AM D3-morphine

Fig. S1 D_3-tracers that were used in competition ED-UPLC/MS/MS

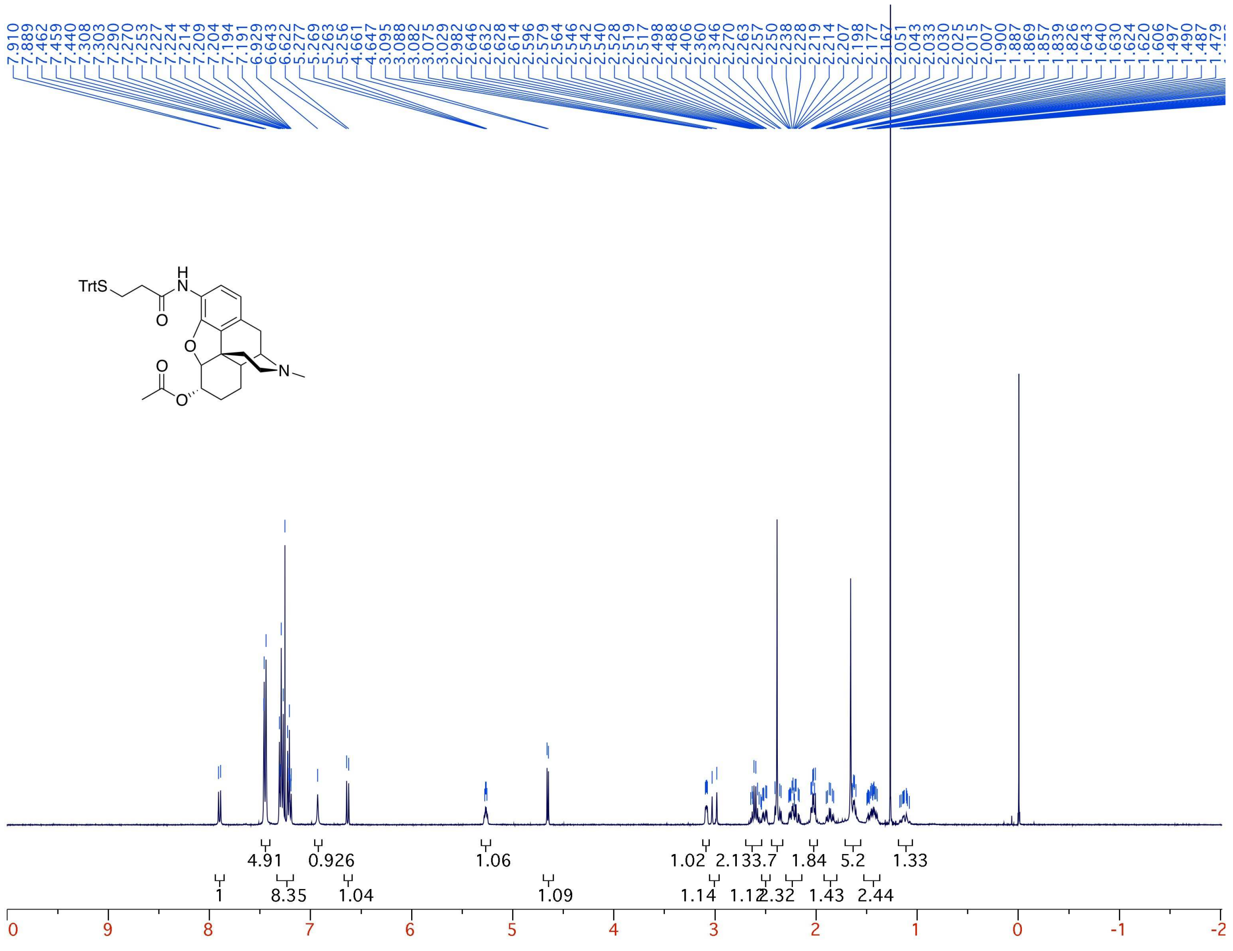

Fig. S2 ^{1}H-NMR of 6-AcMorHap

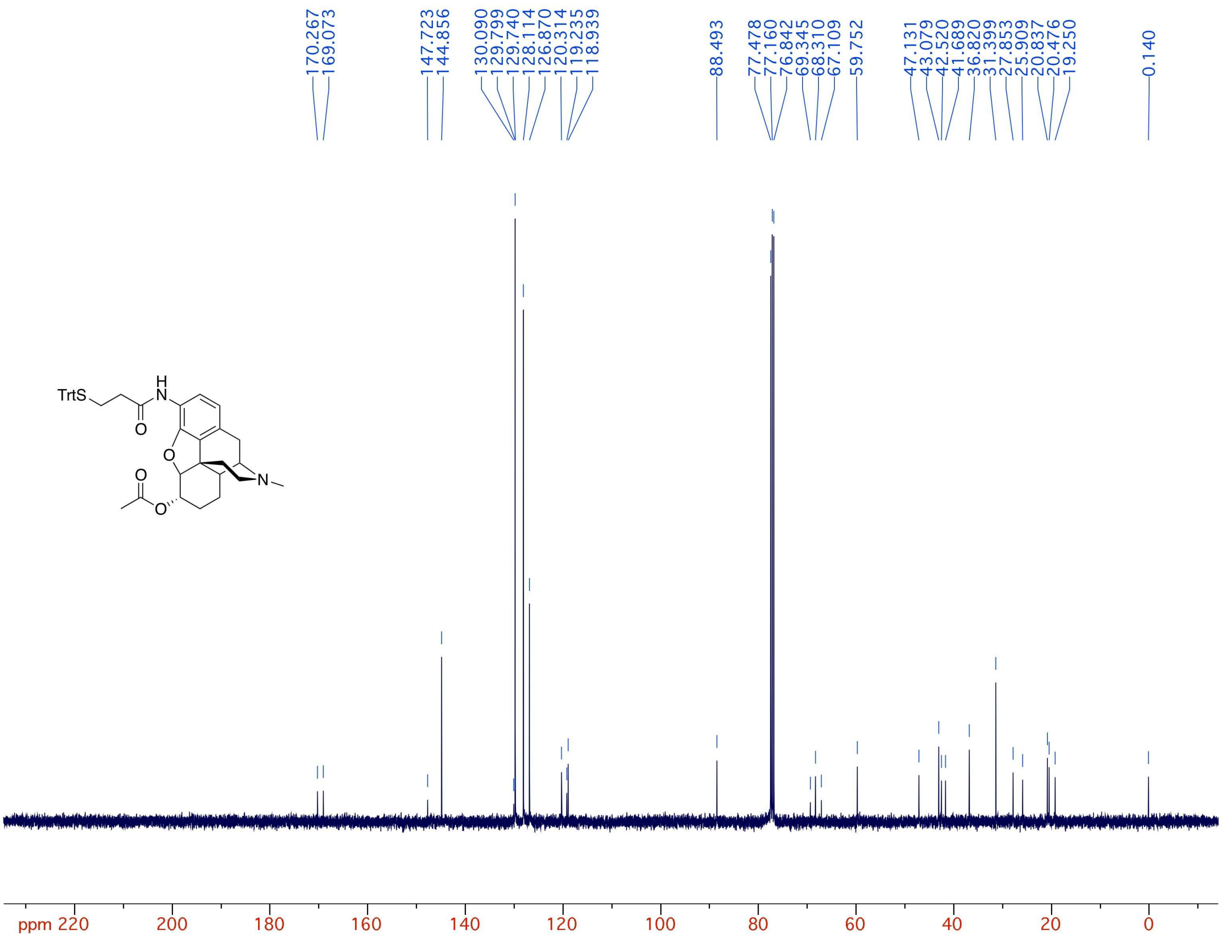

Fig. S3 ^{13}C-NMR of 6-AcMorHap

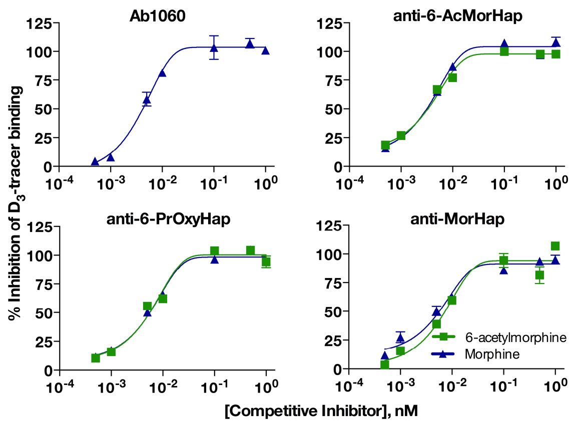

Fig. S4 Competitive inhibition in solution-based ED of 6-AM and morphine to the binding of anti-hapten sera or monoclonal morphine antibody (ab1060) to D_3-tracer (D_3-6-AM or D_3-morphine)

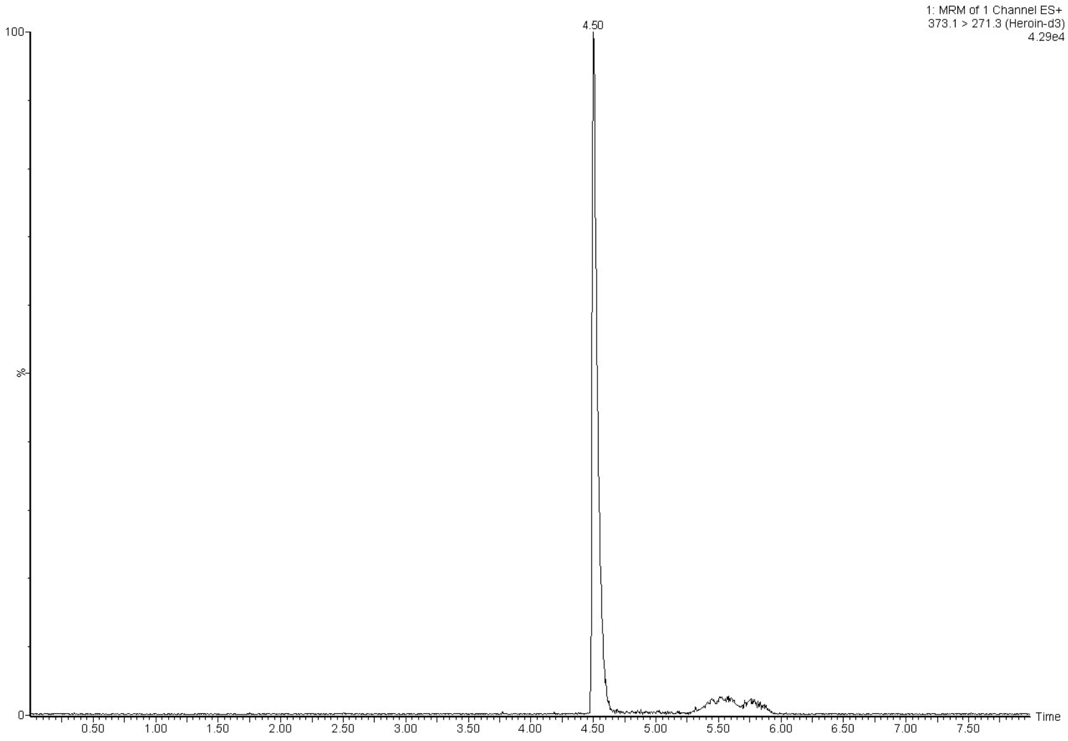

Fig. S5 LC MS spectrum of D_3-heroin

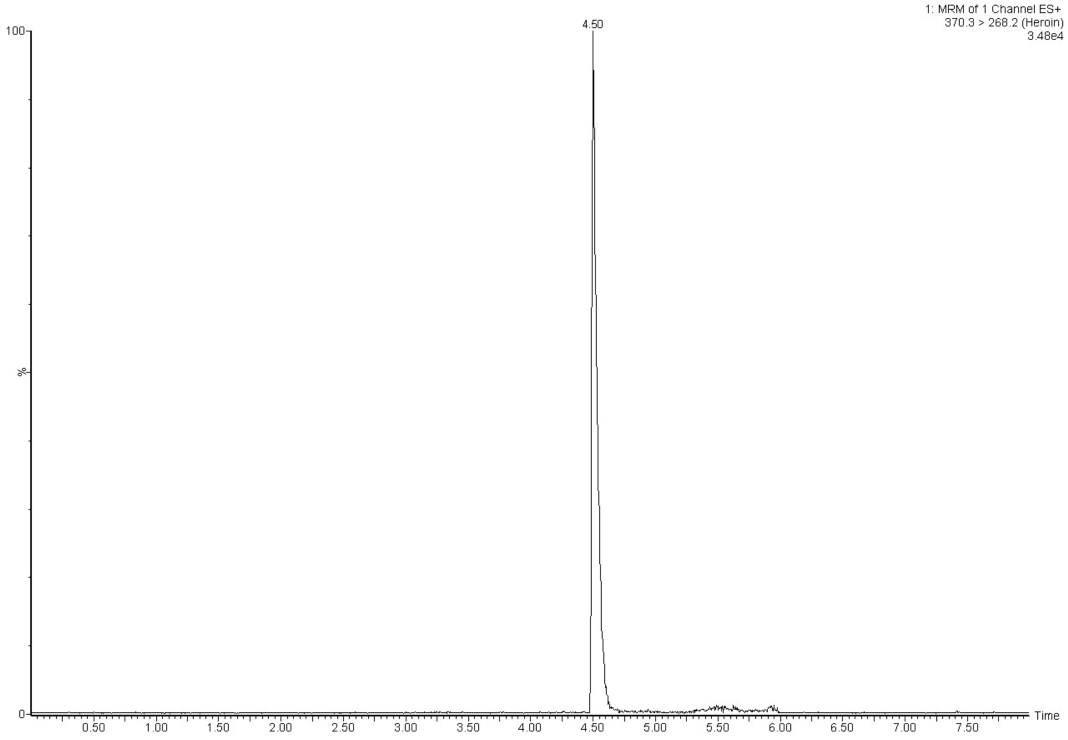

Fig. S6 LC MS spectrum of heroin

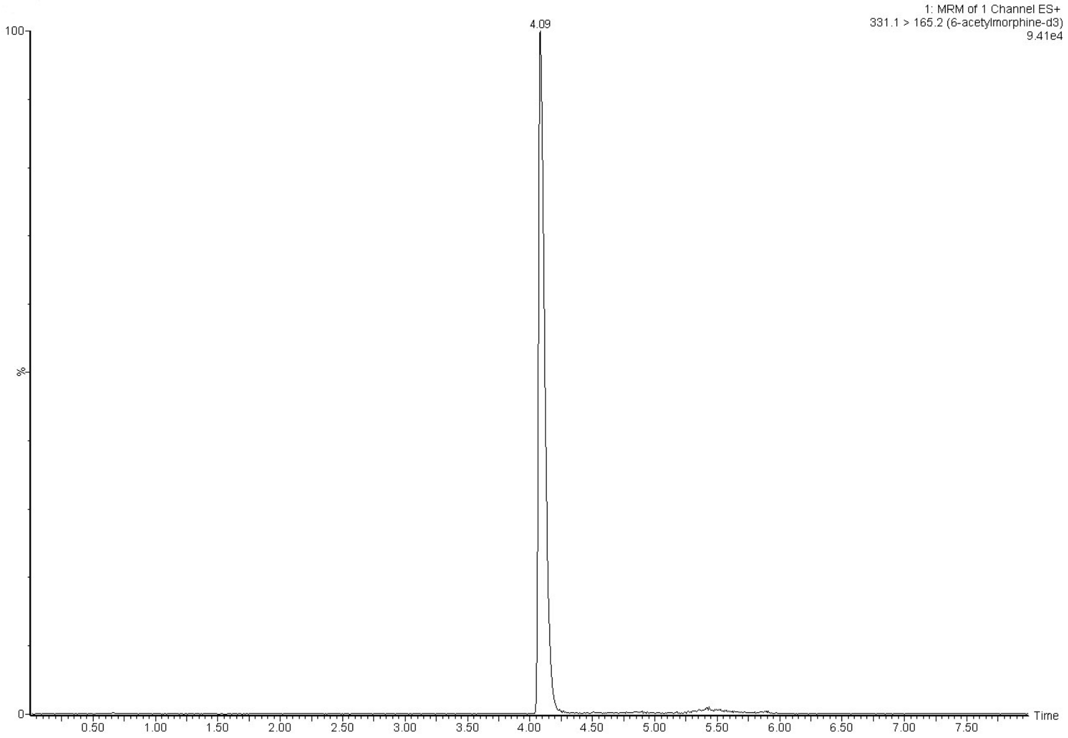

Fig. S7 LC MS spectrum of D_3-6-AM

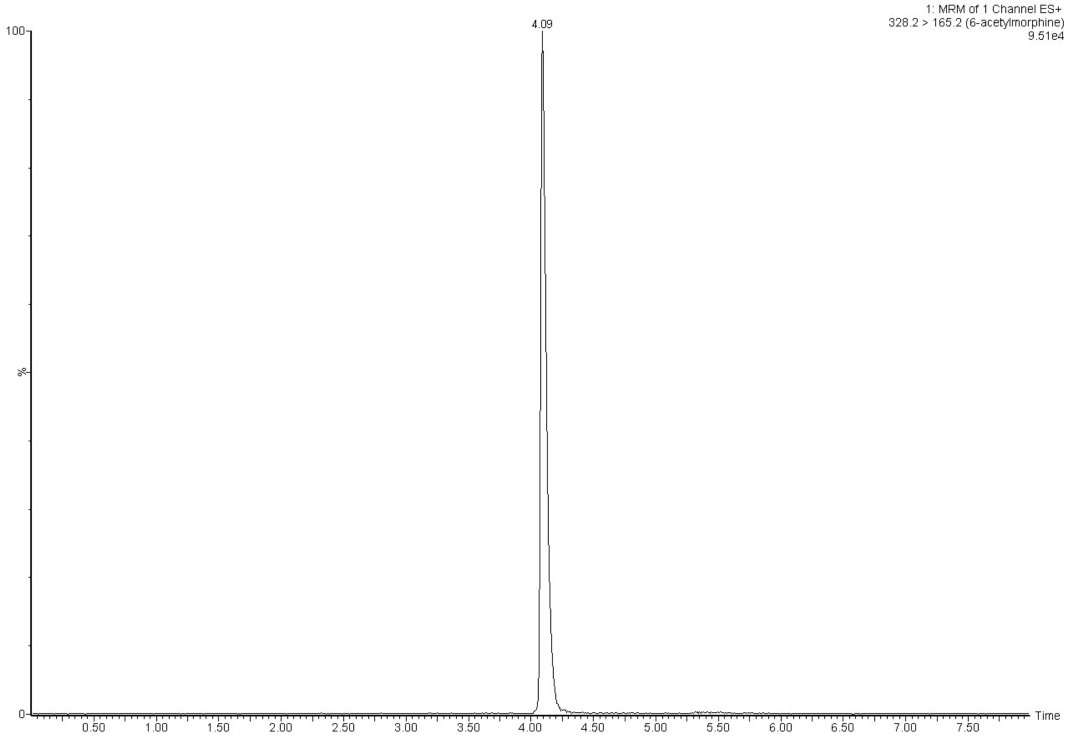

Fig. S8 LC MS spectrum of 6-AM

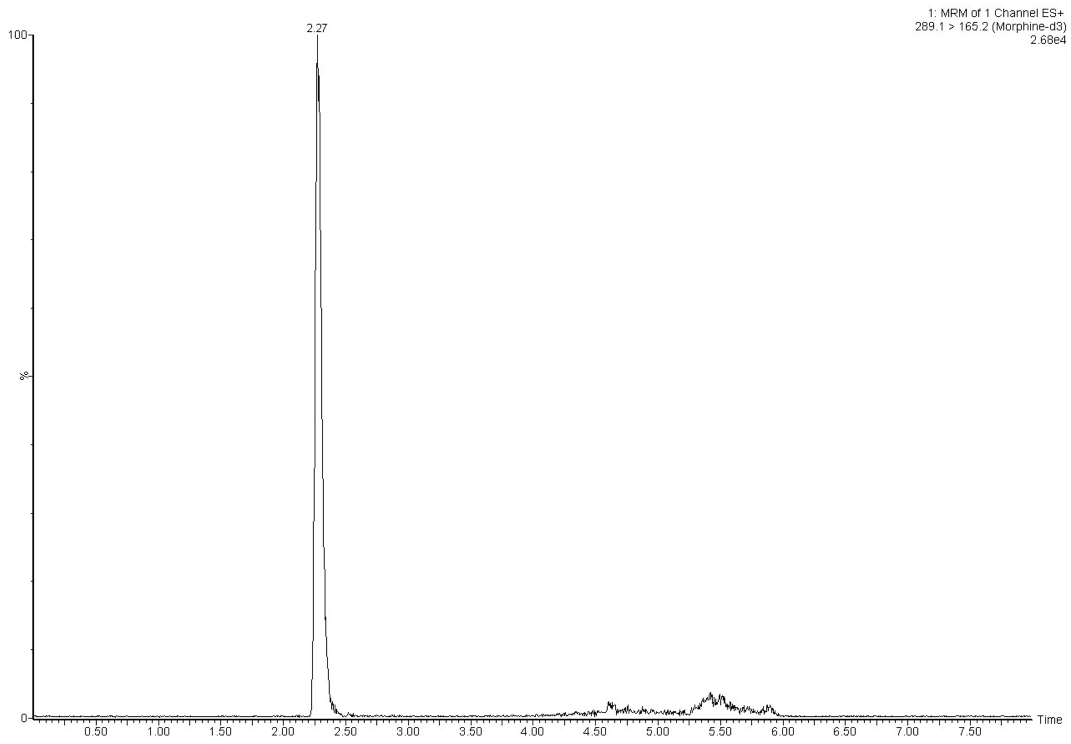

Fig. S9 LC MS spectrum of D_3-morphine

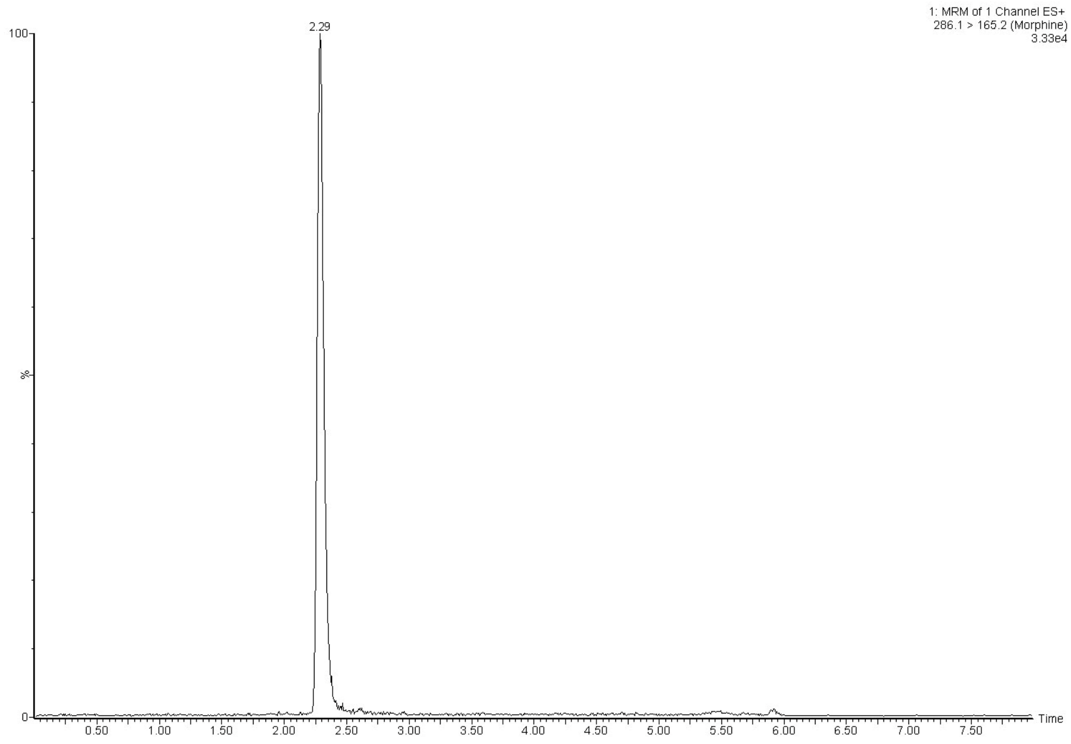

Fig. S10 LC MS spectrum of morphine

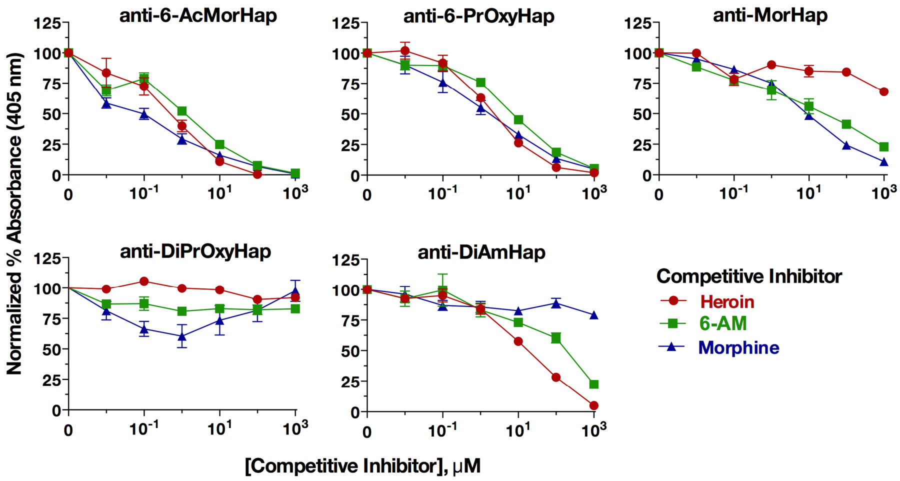

Fig. S11 Competitive inhibition of heroin and its degradation products to the binding of anti-hapten sera to BSA-hapten coated ELISA plates. The graphs show normalized competition curves for anti-6-AcMorHap, 6-PrOxyHap, MorHap, DiAmHap and DiPrOxyHap. Sera from week 9 were pooled from 5 mice per group (same as used for ED). Values are the mean of triplicate determinations ± SEM

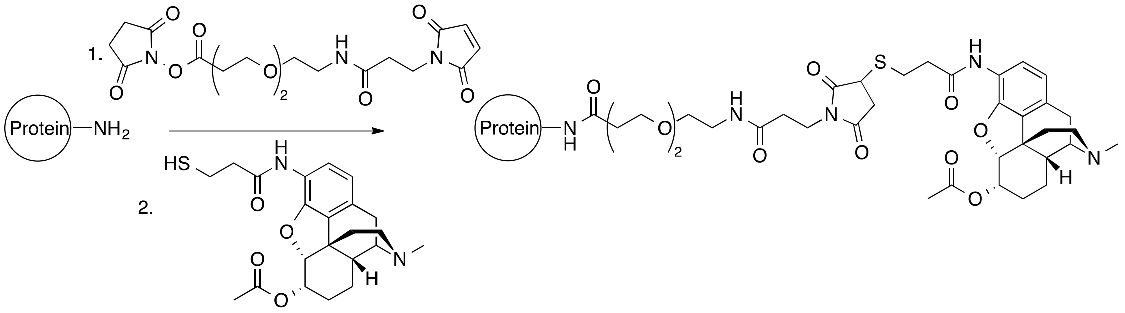

Fig. S12 Synthesis of hapten-bioconjugate (Protein = TT) and ELISA coating agent (Protein = BSA) using maleimide-thiol chemistry

References

1. Alving CR, Matyas GR, Torres O, Jalah R, Beck Z (2014) Adjuvants for vaccines to drugs of abuse and addiction. Vaccine 32:5382-5389.

2. Jalah R, Torres OB, Mayorov AV, Li F, Antoline JF, Jacobson AE, Rice KC, Deschamps JR, Beck Z, Alving CR, Matyas GR (2015) Efficacy, but not antibody titer or affinity, of a heroin hapten conjugate vaccine correlates with increasing hapten densities on tetanus toxoid, but not on CRM197 carriers. Bioconjug Chem 26:1041-1053.

3. Li F, Cheng K, Antoline JF, Iyer MR, Matyas GR, Torres OB, Jalah R, Beck Z, Alving CR, Parrish DA, Deschamps JR, Jacobson AE, Rice KC (2014) Synthesis and immunological effects of heroin vaccines. Org Biomol Chem 12:7211-7232.

4. Matyas GR, Mayorov AV, Rice KC, Jacobson AE, Cheng K, Iyer MR, Li F, Beck Z, Janda KD, Alving CR (2013) Liposomes containing monophosphoryl lipid A: a potent adjuvant system for inducing antibodies to heroin hapten analogs. Vaccine 31:2804-2810.

5. Matyas GR, Rice KC, Cheng K, Li F, Antoline JF, Iyer MR, Jacobson AE, Mayorov AV, Beck Z, Torres OB, Alving CR (2014) Facial recognition of heroin vaccine opiates: Type 1 cross-reactivities of antibodies induced by hydrolytically stable haptenic surrogates of heroin, 6-acetylmorphine, and morphine. Vaccine 32:1473-1479.

6. Muller R (1983) Determination of affinity and specificity of anti-hapten antibodies by competitive radioimmunoassay. Methods in enzymology 92:589-601.

7. Torres OB, Jalah R, Rice KC, Li F, Antoline JF, Iyer MR, Jacobson AE, Boutaghou MN, Alving CR, Matyas GR (2014) Characterization and optimization of heroin hapten-BSA conjugates: method development for the synthesis of reproducible hapten-based vaccines. Anal Bioanal Chem 406:5927-5937.
